# Multi‐Mechanism Collaborative Bionic Fixation Technique Between a Wide Range of Solid Interfaces

**DOI:** 10.1002/advs.202409507

**Published:** 2024-11-26

**Authors:** Shixun Fu, Jun Sun, Zhiyong Hu, Yongjin Zhao, Tianchang Yao, Xipeng Wang, Yuanming Ji, Kai Deng, Keju Ji

**Affiliations:** ^1^ Jiangsu Key Laboratory of Bionic Materials and Equipment Nanjing University of Aeronautics and Astronautics 29 Yudao Street Nanjing 210016 China; ^2^ Shanghai Key Laboratory of Aerospace Intelligent Control Technology Shanghai Aerospace Control Technology Institute Shanghai 201109 China; ^3^ AVIC the First Aircraft Institute Xi'an 710089 China; ^4^ No. 208 Research Institute of China Ordnance Industries Beijing 102202 China

**Keywords:** biomimetic, energy consumption, interfacial adhesion, multi‐mechanism cooperation

## Abstract

For rough surfaces, stable, fast, and repeatable fixation has wide applicability in transportation, fire protection, and other fields. Different rough surfaces present technical challenges for achieving convenient and reliable fixation. Based on the highly adhesive attachment structures of typical organisms, a multi‐mechanism (negative pressure adsorption, mechanical locking, and chemical bonding) cooperative bionic fixation device is proposed. The device is equipped with a suction disc with gradient guide channels, a microneedles friction‐enhancing unit, and fast‐curable UV glue. These components work together to complete the fixation. The detachment work (max. 5.7 and 5.5 J) and pull‐off force (max. 377 and 175 N) are evaluated on sandpaper of different roughness under vertical and horizontal pulling respectively. By analyzing the detachment process and experimental curves, the cooperative principle of the multi‐mechanism is identified. In addition, the microneedles with soft backing at the bionic fixation device bottom improve its adaptability to rough surfaces. The gradient guide channels of the suction disc create Laplace pressure to speed up the UV glue flow and shorten fixation time. Furthermore, its applicability is demonstrated by combining it with monitoring equipment and an adult to attach to rough surfaces.

## Graphical abstract

Based on the highly adhesive attachment structures of typical organisms, a multi‐mechanism (negative pressure adsorption, mechanical locking, and chemical bonding) cooperative bionic fixation device is proposed. The device adheres to a wide range of rough surfaces. Moreover, the most suitable type of fixation can be selected according to the application environment and the forces applied.

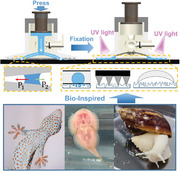

## 1 Introduction

Certain fields, such as civil transportation, surveillance, firefighting, and national defense, require equipment to be stably and fast placed on rough surfaces. However, the varied topographical characteristics of surfaces, including roughness, material, and curvature, challenge reliable, rapid, and consistent equipment deployment. For instance, the negative pressure adsorption sucker has specific surface finish requirements for attachment. Negative pressure fans consume high energy and generate noise. Hooks and claws may fail on smooth surfaces and damage sensitive areas. The high voltage demands of electrostatic adsorption and the specificity of magnetic adsorption to surface materials also present challenges. So, it is challenging to be able to attach to a wide range of rough surfaces through a single device.

Reliable attachment and fixation capabilities are demonstrated by many organisms in their living environments. Most organisms use two or more mechanisms to achieve stable and controllable attachment and fixation, adapting to the complex surface characteristics in nature. For example, geckos rely on van der Waals forces generated by microfibers on their feet and mechanical locking between their pointed claws and surfaces for climbing.^[^ ^1^, ^2^, ^3^, ^4^ ^]^ Snails use mucus adhesion and foot suction to adhere to surfaces and move by controlling the mucus state and the foot muscles.^[^ ^5^, ^6^, ^7^, ^8^ ^]^ Tree frogs rely on capillary adhesion generated by the toe pads and hydrodynamic adhesion to adhere to moist environments, with van der Waals forces and mechanical locking also believed to contribute to adhesion.^[^ ^9^, ^10^, ^11^, ^12^, ^13^, ^14^ ^]^ Hill stream fishes use the suction of their discs to adhere to rocks, while the layered fluff on the periphery of their discs increases friction on irregular surfaces.^[^ ^15^, ^16^, ^17^, ^18^, ^19^ ^]^ Some insects, such as beetles, flies, ants, and cockroaches use claws with adhesive pads that are interlocked to surfaces with both van der Waals forces to produce strong adhesion.^[^ ^20^, ^21^, ^22^, ^23^ ^]^

Recently, multiple attachment mechanisms in organisms were studied for biomimetic attachment design. For smoother surfaces, an adhesive material designed based on octopus's suction cup protrusions showed strong adhesion to silicon wafers and glass.^[^ ^24^ ^]^ Protein‐coated suction cup devices inspired by octopus and mussels achieve good reversible adhesion to silicon wafers and aluminum blocks in dry and wet environments.^[^ ^25^ ^]^ For medium roughness surfaces, an artificial attachment device mimicking insect‐hooked claws and adhesive pads increased the range of coping with surface protrusions.^[^ ^26^ ^]^ A controllable adhesion structure inspired by octopus suction cups attached reliably on non‐ideal surfaces under low pre‐pressure and was capable of maneuvering various underwater objects.^[^ ^27^ ^]^ For high‐roughness surfaces, an multi‐material biomimetic remora disc with carbon fiber microneedles inside can be attached to multiple types of surfaces.^[^ ^28^, ^29^ ^]^ Inspired by hill stream fishes, a suction disc with edge microstructure which form a cooperative effect with the disc cavity to enhance the adhesion adhered to the substrate of different roughness.^[^ ^30^ ^]^ In addition, some comprehensive bionic attachment structures that perform well on surfaces with different roughness. A multi‐mechanism bionic adhesion structure designed by biological cushioning and foot structure can achieve good adhesion in collision and vibration environments.^[^ ^31^ ^]^ A highly adaptive soft adhesion device attached robustly to dry and wet surfaces with diverse morphologies, allowing conformal attachment on curved and soft objects with high roughness.^[^ ^32^ ^]^ These adhesion structures inspired by biological attachment modes can provide adhesion and be applied on multi‐material and multi‐roughness surfaces. However, further studies at enhancing the efficacy of biomimetic adhesive systems combined with various mechanisms, offer increased resistance against detachment during adherence on rough surfaces while enduring multi‐directional forces.

Inspired by the attachable and fixable mechanisms of organisms such as geckos, snails, and hill stream fishes, a device proposed is based on an end‐expanded suction disc structure with tapered, large‐arc‐shaped channels, a microneedles friction‐enhancing unit, and fast‐curing UV glue. The device exhausts air from the suction disc and the rough surface by the UV glue flowing into the suction disc guide channel. After UV glue is cured, there is mechanical locking and chemical bonding between the suction disc and the cured UV glue, negative pressure adsorption between the suction disc and the rough surface, and mechanical locking between the microneedles and the rough surface. These three mechanisms work cooperatively allowing the device to achieve stable fixation on a wide range of rough surfaces from tangential friction to normal adhesion in the whole space. The adaptability of the microneedles to rough surfaces was explored by altering the hardness of the microneedles backing. Additionally, the driving effect of the Laplace pressure in the radial gradient guide channels of the suction disc was also investigated.^[^ ^33^ ^]^ Subsequently, the bionic fixation device was used as the application in combining equipment. After applying a load to the device, it was able to attach to rough surfaces.

## 2 Results and Discussion

### 2.1 Fabrication of Bionic Fixation Device

The manufacturing process of the suction disc uses 3D printer (FUNMAT PRO 410) to print out the upper, middle, and lower molds of the suction disc's molds. The suction disc's upper, middle, and lower molds are secured by bolting. A liquid silicone with a Shore hardness of 40 is weighed 120 g and mixed, stirred fully, vacuumed, and poured into the middle and lower molds. Lower and middle molds are locked by bolts. Afterward, 50 g of silicone with a Shore hardness of 40 is prepared in the same way and poured into the upper mold and between the middle and lower molds. The upper mold and the other molds are positioned and locked by the small cylinders on the side. The filled molds are then placed in an oven, where the temperature is adjusted to 45 °C and left for 2 h to cure. Finally separated from the molds to obtain the silicone suction disc (Figures S2 and S7, Supporting Information). Hard backing microneedles were made of aluminum alloy 6061 through Computerized Numerical Control (CNC). The process of making soft‐backed microneedles involves first 3D printing a mold with many small holes at the bottom, and laser processing the gauze tape to create the corresponding holes. After the tape is attached to the mold according to the corresponding holes, the microneedles are embedded into the holes of the tape and the mold. In the same way, as described above, 10 g of silicone gel with a Shore hardness of 5 was prepared and poured into the mold. The filled mold is then placed in an oven, where the temperature is adjusted to 45 °C and left for 2 h to cure (Figure S5, Supporting Information). The remaining components of the device were fabricated from Teflon, a material known for its low surface energy which ensures the unobstructed flow of UV glue. CNC was also chosen as the machining method for these components. Complex structural components such as the upper body and the lower body are machined by five‐axis machining centers, and the rest of the components are machined by three‐axis CNC milling machines.

### 2.2 Bioinspired Design of Bionic Fixation Device

**Figure 1:**
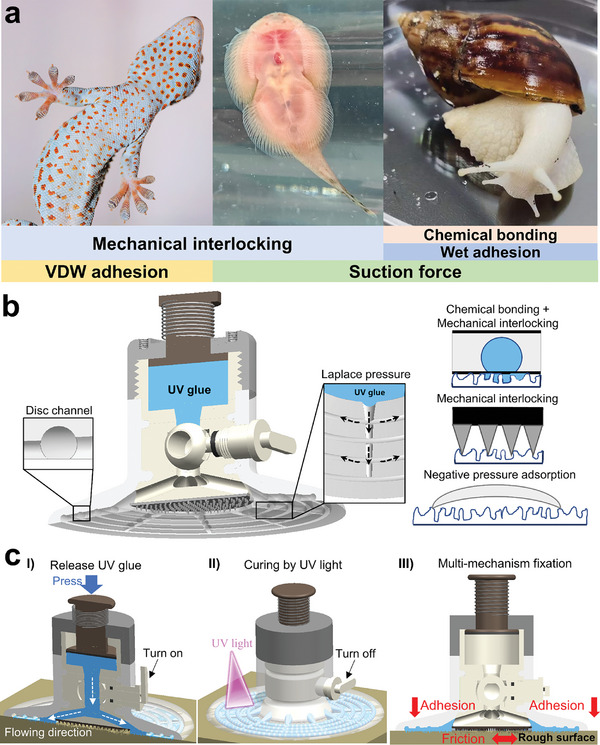
Schematic bionic principle. a) Typical multi‐mechanistic organisms. b) Schematic of the multi‐mechanism cooperative bionic fixation device. c) Functional realization process of the multi‐mechanism cooperative bionic fixation device.

As shown in **Figure** **1**a, the multi‐mechanism cooperative bionic fixation device was based on the key morphological features of hill stream fishes (soft disc, microneedles), snails (flexible ventral foot, mucus), and geckos (claws). The device consists of 16 components, of which the suction disc, microneedles, and UV glue are the most important parts (Figure 1b; Figure S1, Supporting Information). The device function is achieved in the way shown in Figure 1c. First, the device is pre‐loaded so that it adheres well to the rough surfaces. Then turn the switch knob 90°, and the switch knob drives the stem to rotate, the stem drives the ball to rotate, so the valve of the ball valve is opened. Press the push rod to push the UV glue out of the upper body. After that, UV glue flows into the corresponding suction disc's guide channels through several circular arrays of cylindrical holes in the lower body. UV glue overflows under the action of Laplace pressure for autonomous transportation to penetrate between the micropores on the rough surface. Rotate the switch knob, close the ball valve, use UV light to expose, and wait for UV glue to be cured to complete the fixation. Because UV glue infiltrates and fills the pores of rough surfaces to discharge air, a suction disc can be formed around the center of the multiple sealing ways to make the device even if adhered to a rough surface still has the effect of negative pressure adsorption. Chemical bonding and mechanical locking between the UV glue cured and the large arc‐shaped guide groove of the suction disc. The microneedles at the bottom of the device are stuck with the concave and convex rough surfaces to produce a mechanical lock effect. Thus, the bionic fixation device has the cooperative effect of negative pressure adsorption, mechanical locking, and chemical bonding.

### 2.3 Detachment Work and Pull‐off Force Results of Bionic Fixation Device

**Figure 2:**
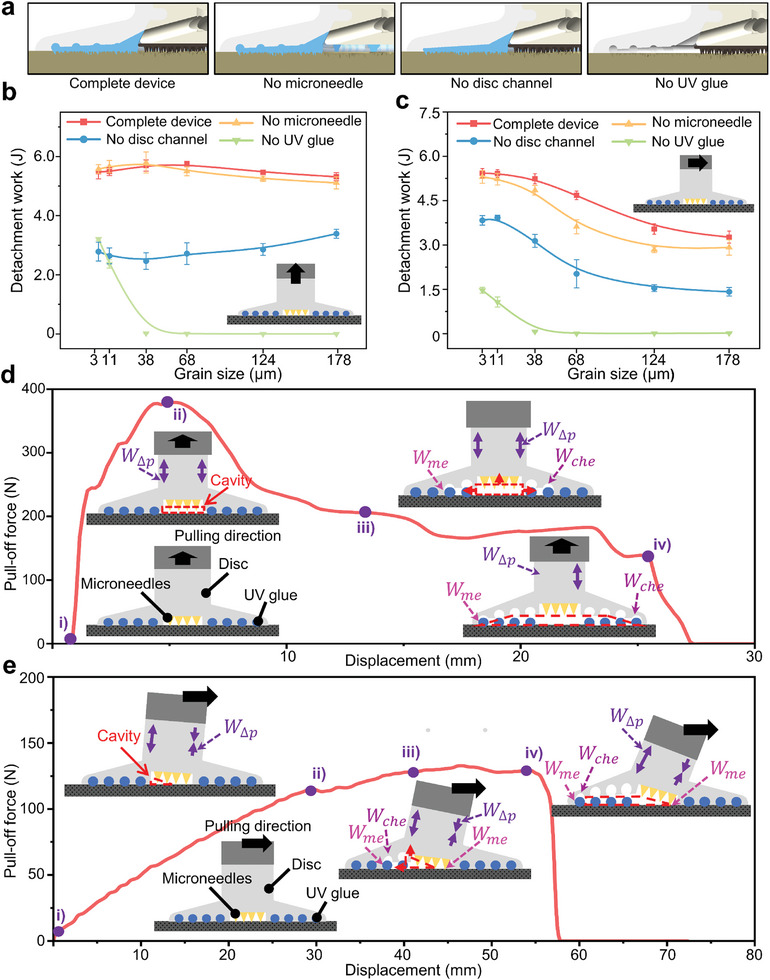
Measurements of detachment work for different devices and different conditions. a) Schematics of four different devices, complete device, no microneedle device, no disc channel device, no UV glue device. b,c) Detachment work for different devices under varying grain sizes: b) vertical, c) horizontal. d,e) Pull‐off force‐time curves (grain size ≈178 µm) and schematic of the desorption process for the complete device: d) vertical, e) horizontal.

To study the cooperative benefits of multi‐mechanism, several bionic fixation devices with different structures were fabricated, including a complete device, no microneedle device, no disc channel device, and no UV glue device as shown in **Figure** **2**a. Experimentally tested the differences in the desorption process for each device when detached from surfaces of different roughness (Figure S6, Supporting Information). The ability of these bionic fixation devices to resist desorption can be reflected by detachment work *W*_d_, which is the energy consumed by the device to detach from rough surfaces. The detachment work is defined as the area under the force–displacement curve, from initial displacement to failure.^[^ ^5^, ^34^ ^]^

(1) $$W_{d}=\int F\;d\delta =W_{\Delta P}+W_{che}+W_{me}$$

where *F* is pull‐off force and δ is displacement. It includes the energy *W*_Δ*P*_ consumed by the deformation of the suction disc due to negative pressure adsorption, the energy *W*_che_ required by chemical bonding between the suction disc and cured UV glue, and the energy *W*_me_ consumed by large‐arc‐shaped channels of the suction disc detach from cured UV glue and mechanical locking between microneedles and surfaces (Figure 2d,e).

When devices were vertically pulled and detached from different rough surfaces, mostly the detachment work of the complete device was the largest (Figure 2b), with maximum detachment work (≈5.7 J) and pull‐off force (≈377 N), which is ≈140 times of the gravity of the complete device (≈2.69 N). The vertical pulling desorption process of the complete device can be divided into three stages (first stage between Figure 2d‐i,ii, between Figure 2d‐ii,iv is the second stage, and after Figure 2d‐iv is the third stage). The first stage involves the detachment of the innermost guide channel from the cured UV glue, resulting in the formation of a cavity between them. So there exists a pressure difference concerning the external environment, and the pull‐off force increased rapidly due to tensile deformation in the upper part of the suction disc until reaching its maximum (Figure 2d‐ii). In the second stage, after the device reaches the maximum pull‐off force, the cavity expands in all directions, and the rest of the suction disc's guide channels are gradually detached from the cured UV glue. Each time a part of the guide channels is detached, the tensile deformation of the suction disc will be smaller (Figure 2d‐iii). The second stage of the curve demonstrates that after a slight increase in pull‐off force, it either maintained a constant value or slightly decreased. The third stage is the complete detachment of the device from the rough surface, i.e., the outermost cycle of the suction disc's guide channel is detached from the cured UV glue and the pull‐off force decreases suddenly to 0 N (Figure 2d‐iv).

When devices were horizontally pulled to detach on different rough surfaces, the detachment work of the complete device was the largest (Figure 2c), with maximum detachment work (≈5.5J) and detachment force (≈175 N), which is ≈69 times the gravity of the complete device. The complete device horizontal pulled desorption process is also divided into three stages (the first stage between Figure 2e‐i,ii, the second stage between Figure 2e‐ii,iv, and the third stage after Figure 2e‐iv). In the first stage after the device is pulled horizontally, the bottom guide channel on one side of the suction disc (pulled side) will have a tendency to detach from the cured UV glue, and the other side of the suction disc (stressed side) will be squeezed and tightly adhered to the cured UV glue. A pressure difference is created in the cavity formed between the device and the cured UV glue. The pull‐off force will continue to increase with the stretching of the upper part of the suction disc and the mechanical locking of the microneedles with the substrate. The curve shows a larger slope in this phase, and the pull‐off force reaches a larger value in a short time (Figure 2e‐ii). In the second stage, the cavity expands toward the pulled side, and the guide channels of the pulled side are gradually detached. The device tilts toward the pulling direction, and the tensile deformation on the suction disc pulling side is slightly weakened (Figure 2e‐iii). Unlike the second stage curve of the vertical pulling, the curve of the horizontal pulling does not show a decrease in the pull‐off force as the suction disc pulling retracts. The reason is that when the device is pulled horizontally, the microneedles provide part of the force to resist the tilting of the device, and when the deformation of the pulled side of the suction disc is weakened, the stressed side generates the force to recover the deformation at the same time. Therefore, three conditions of weakened deformation on the pulled side, continued pressure on the stressed side, and mechanical locking of the microneedles with the substrate existed at the same time, the curves of the second stage showed no change or a small increase in the pull‐off force (Figure 2e). In the third stage, the outermost ring of the suction disc's guide channel is detached from the cured UV glue. The whole device was completely tilted toward the direction of pulling, meanwhile the force value decreased to 0 N (Figure 2e‐iv).

**Figure 3:**
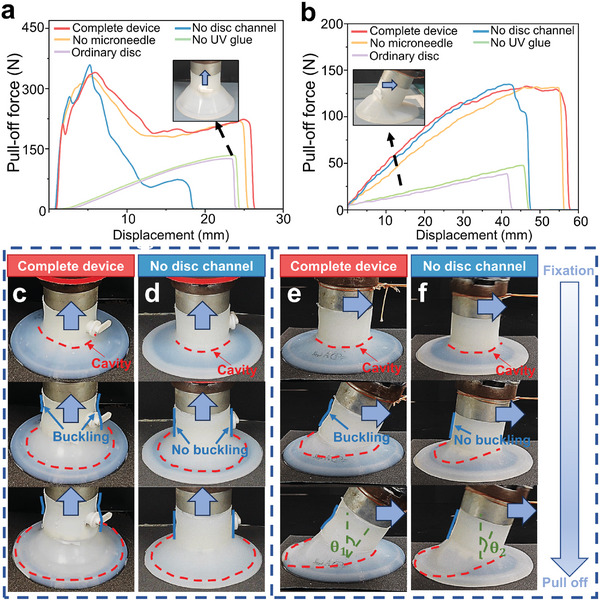
Desorption process of different devices. a,b) Pull‐off force‐time curves for different devices and different directions (grain size ≈ 11 µm): a) vertical, b) horizontal. c–f) Differences in the desorption process between the complete device and the no disc channel device at the same desorption speed: c) complete device, vertical, d) no disc channel device, vertical, e) complete device, horizontal, f) no disc channel device, horizontal.

The pull‐off force of the no UV glue device in vertical pulling is the smallest (**Figure** **3**a; Figure S9a, Supporting Information). As shown in Figure 3a, its pull‐off force‐time curve and detachment process are similar to ordinary suction disc, which indicates that the negative pressure adsorption of the suction disc plays a major role in the process of the no UV glue device vertical pulling. Therefore, with the increase of grain sizes of the sandpaper, the sealing effect of the suction disc is weakened until disappears, so the pull‐off force and the detachment work are decreased accordingly. For the no microneedle device and the complete device, their experimental curves are similar, but there will still be some small differences (Figure 2b). There are probably two reasons for these, one is a small amount of UV glue formed a chemical bond with the substrate and some of the microneedles, so that some of the microneedles and the substrate were bonded together by UV glue. The other is the absence of microneedles on the bottom of no microneedle device, where a small amount of uncured UV glue will remain on the bottom and result in the inability of the UV glue to fill all the guide channels of the suction disc (Figure S11, Supporting Information). The combination of these two reasons leads to small differences in the mechanical properties of a complete device and no microneedle device. No disc channel device compared with the complete device, the structural difference is that the suction disc's guide channel is only the innermost circle as shown in Figure 2a. In the process of no disc channel device detachment, the first stage is similar to the complete device, which is the formation of a cavity between the innermost guide channel and the cured UV glue. The suction disc is deformed by stretching to achieve maximum pull‐off force. Therefore, the pull‐off force and the displacement to reach maximum pull‐off force of the no disc channel device are similar to complete device (Figure 3a). After that, the cavity expands rapidly in all directions until detached (Figure 3d). The suction upper part remains stable and cylindrical without buckling during vertical puling of the no disc channel device (Figure 3d). While the complete device is pulled vertically, due to the formation of mechanical locking between the suction disc's guide channels and the cured UV glue, the upper part of the suction disc is stretched and squeezed against the device's body to a greater extent, resulting in buckling (Figure 3c). The no disc channel device detaches faster from the surface of the cured UV glue (compared to the complete device) and the pull‐off force is continuously decreased. The no disc channel device takes longer to reduce the pull‐off force to zero (compared to no UV glue device) due to the greater vacuum of the no disc channel device and the chemical bonding between the suction disc and the cured UV glue.

As shown in Figure 3b, when each device is pulled horizontally, the complete device compared with no microneedle device has mechanical locking between the microneedles and the substrate that provides friction, so that the device better resists detachment. Compared with no UV glue device, the existence of UV glue makes the air between the suction disc and rough surfaces discharged to achieve the sealing effect. Compared with no disc channel device, the locking and bonding of the suction disc guide channel with the cured UV glue make it reach the maximum pull‐off force without rapid desorption resulting in a sharp decrease in force value (Figure 3e,f) and the upper part of the suction disc also produces buckling phenomenon. The tilt angle *θ*_1_ of the complete device is significantly larger than that *θ*_2_ of the no disc channel device, which also proves the multi‐mechanism cooperation of the complete device. It was also observed that the horizontal detachment work of the devices decreased with the increase of surface roughness due to the gradual difficulty of filling the guide channels with UV glue, making the interaction between the outermost ring of the suction disc and the UV glue weaker.

In summary, both detachment work‐grain size and the pull‐off force‐time curves demonstrate that the complete device has the best fixation effect on different rough surfaces. By comparing the desorption process of bionic fixation devices with different structures, the negative pressure adsorption mechanism playing a decisive role in the maximum pull‐off force (The UV glue flows into the rough surface to discharges the air, relying on the suction disc as carrier to assist the suction disc can form a negative pressure adsorption on rough surfaces.) was proved. The mechanical locking and chemical bonding between the suction disc's guide channels and the cured UV glue as well as between the microneedles and the substrate slows down the decreased of the pull‐off force and increases the time of desorption (Figures 2 and 3; Figure S10, Supporting Information). This strategy of multi‐mechanism cooperation to increase energy consumption effectively enhances the anti‐desorption ability of the bionic fixation device, which can make the device better resistant to external loads.

### 2.4 Friction of Microneedles Backing Hardness

**Figure 4:**
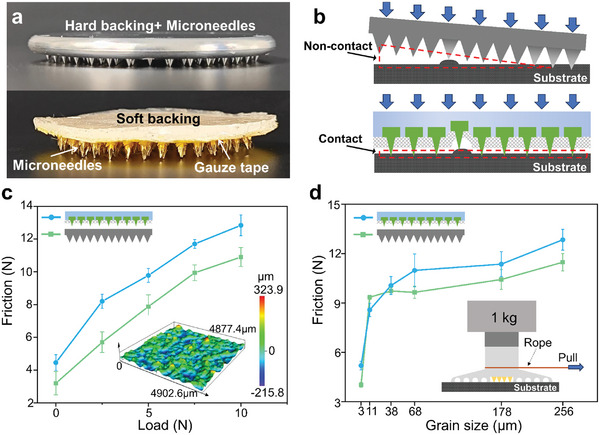
a) Schematics of hard‐backed microneedles and soft‐backed microneedles. b) Schematic of microneedles contact with substrate. c) Friction‐load (grain size ≈ 256 µm) and d) friction‐grain size curves for a device equipped with two different microneedles (load = 10 N).

For metal microneedles, the hardness of the microneedles has little effect on the friction because the Young's modulus is at the GPa level and the deformations generated by the force are small (Figure S13, Supporting Information). So, it is more important to explore the modulus of elasticity of the microneedles backing (*E*). The effect of microneedles backing on the bionic fixation device is further investigated after horizontal pulling under different rough surfaces and loads. Meanwhile, the bionic fixation device in the absence of negative pressure adsorption and UV glue only uses the friction of the microneedles to achieve fixation to prove the role of the microneedle's mechanical locking effect. The materials of two different microneedles backings are metal (hard backing) and hardness 5 silicone (soft backing). As shown in **Figure** **4**a, the hard‐backed microneedles are CNC machined as a whole while the soft‐backed microneedles are manufactured by casting (Figure S5, Supporting Information). The soft‐backed microneedles can be divided into three parts. The upper layer is silicone. The middle layer is gauze tape and this layer has a function of bonding the silicone layer and fixing the microneedles between the silicone layer and the gauze tape. The lower layer is the microneedles, which are also made of metal.

Experiments were conducted to measure the friction provided by two microneedles when the no‐UV glue device was pulled horizontally at different load (*F_load_*) and on surfaces of different roughness (*R*_a_). The friction *F*_f_ provided by the soft‐backed microneedles can be given by Equation (2) (see the Supporting Information for more details)

(2) $$F_{f}=\mu n\int F_{n}d\Delta h=\mu \left(F_{\mathrm{load}}+\frac{n^{2}\;\mathrm{AE}\;R_{a}}{H}\right)$$

where *µ* is the coefficient of static friction between the microneedle material and the rough surface; *A* is the cross‐sectional area of the microneedle end; *H* is the thickness of the backing;*n* is the number of microneedles. From Equation (2) and Figure 4, it can be seen that the friction increases with increasing load and roughness. Microneedles must be lifted over the convexity of the surface before they can be pulled. With load increasing, the force required to lift the microneedles is greater, at the same time, the area of the microneedles locked to the rough surface after bearing the load is larger. So, more force is needed to pull the microneedles (Figure 4c). Similarly, as the surface roughness increases, the size of the unevenness of the surface increases along with the roughness, the microneedle's tips are blocked more, and the contact area is larger, so the force required to pull the microneedles is greater (Figure 4d). From Equation (2), it can be seen that the higher the *E* of the backing, the higher the friction provided by the microneedles. The modulus of elasticity of the hard backing is much higher than the soft backing, but the friction is reduced. This is because there is a maximum value *E_max_*  =  *F_load_* *H*/*nAR_a_*. When *E* exceeds this value, the backing cannot deform well enough to ensure that the microneedles forms effective contact with the rough surface. As can be seen from Figure 4b, when the hard‐backed microneedles are pulled horizontally, they will be tilted when they encounter a larger size concave and convex place on the surface. The microneedles will be lifted as a whole, only a small part of the microneedles is in effective contact with the surface. When the soft‐backed microneedle encounters rough surface convexity, the soft backing has a cushioning effect that allows the microneedles to individually generate near‐vertical displacements without causing the entire microneedles to be lifted. The cushioning effect of the soft backing ensures effective contact between the microneedles and the rough surface, allowing the microneedles to provide more friction.

### 2.5 Laplace Pressure in Guide Channels of Suction Disc

**Figure 5:**
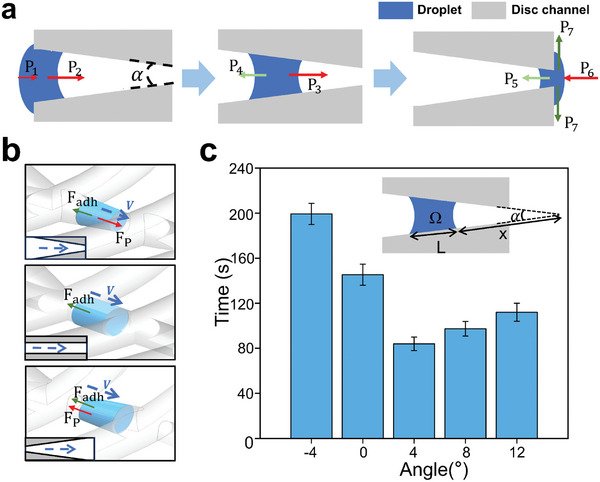
a) Schematic illustrations of UV glue transport mechanism. b) Force analysis and c) full flow time of UV glue in different suction discs.

To realize the directional autonomous transport of UV glue and complete the infiltration and filling of rough surfaces in a short period, semi‐open guide channels with a tapered gradient at the suction disc bottom are designed. The specific principle of Laplace pressure can be explained by **Figure** **5**a, when a droplet is in contact with a tapered gradient guide channel, it is subjected to Laplace pressure *P* = *γ/r*, where *γ* is the liquid prepolymer surface tension; *r* is the radius of curvature of the droplet's surface.^[^ ^35^ ^]^ When a droplet enters through the large opening of the guide channel, the guide channel generates Laplace pressure *P*_1_ + *P*_2_ on the droplet, where *P*_1_ is 0; *P*_2_ = *γ/r*_2_. At the same time, the presence of the Laplace pressure *P_7_ > P_6_ + P*_5_ at the small opening of the guide channel ensures that the droplet does not pass through the guide channel and prevents it from flowing in the opposite direction. The autonomously driven transport of the liquid can be realized by a simple surface‐wetting modulation. When the droplet is in the area between the guide channel, it is subjected to the driving force *F*_p_ = (P_3_ – P_4_) S = (γ / r_3_ – γ / r_4_) S > 0, where *S* is the cross‐sectional area of the droplet.

To explore the suction disc guide channel angle that drives the fastest flow of UV glue, five types of suction discs with radial guide channel of different angles (*α*) of −4°, 0°, 4°, 8°, 12° are fabricated. Differences in autonomous flow‐conducting ability are characterized by the time of delivery of UV glue. As shown in Figure 5c, as the angle of the guide channel increases from −4° to 12°, the UV glue flow time decreases and then increases under room temperature of 35 °C. In reality, the UV glue only partially wets the solid (contact angle between UV glue and suction disc is 64°, Figure S14, Supporting Information), its droplets is also subjected to adhesion resistance *F_adh_* (*F_adh_* = *γ* *W*Δcos *θ*, Δcos *θ* = cos *θ*_*r*_  − cos *θ*_*a*_, *W* is the length of the advancing contact line, *θ*_*a*_ is the advancing contact angle, *θ*_*r*_ is the receding contact angle)^[^ ^33^ ^]^ when flowing in the guide channel. For the three types of suction discs with angles greater than 0°, 0°, and less than 0°, the combined forces on the droplet in the guide channel are *F_p –_ F_adh_*, *– F_adh_*, *–* (*F_p_ + F_adh_*) respectively (Figure 5b). This is the reason for the reduced UV glue flow time in suction discs with an angle of −4° to 4°.

The Laplace driving force is explored in more detail to analyze suction discs with angles greater than 0°. As shown in Figure 5b, the Laplace driving force can be expressed as (see the Supporting Information for more details)

(3) $$F_{p}=\int \gamma \cos\theta /\alpha x^{2}\cdot d\Omega =\frac{8\gamma \Omega^{2}}{\pi \alpha^{3}x^{5}}\;\;\cos\theta$$

where *Ω*  = π*L* *α* ^2^ *x* ^2^/4  is the volume of the droplet; *x* is the distance from the droplet to the top. It can be seen from Equation (3) that the Laplace driving force decreases with increasing angle. Therefore, choosing the suction disc with an angle of 4° is most efficient for the flow of UV glue. For suction discs with angle greater than 0°, the combined force direction of the partially wetted liquid UV glue inside the suction disc is always opposite to the direction of UV glue motion, which means the droplet is subjected to a combined force that is still resistance. Experiments with UV glue with different surface tensions flowing full of suction disc guide channels also proved this conclusion (Figure S17, Supporting Information). Because the droplet flows down from the device's upper body, the gravitational potential energy is converted into kinetic energy and has an initial velocity *v*, so the droplet can flow to the outermost ring of the suction disc's guide channel when it is subjected to resistance.

### 2.6 Applications for Bionic Fixation Device

**Figure 6:**
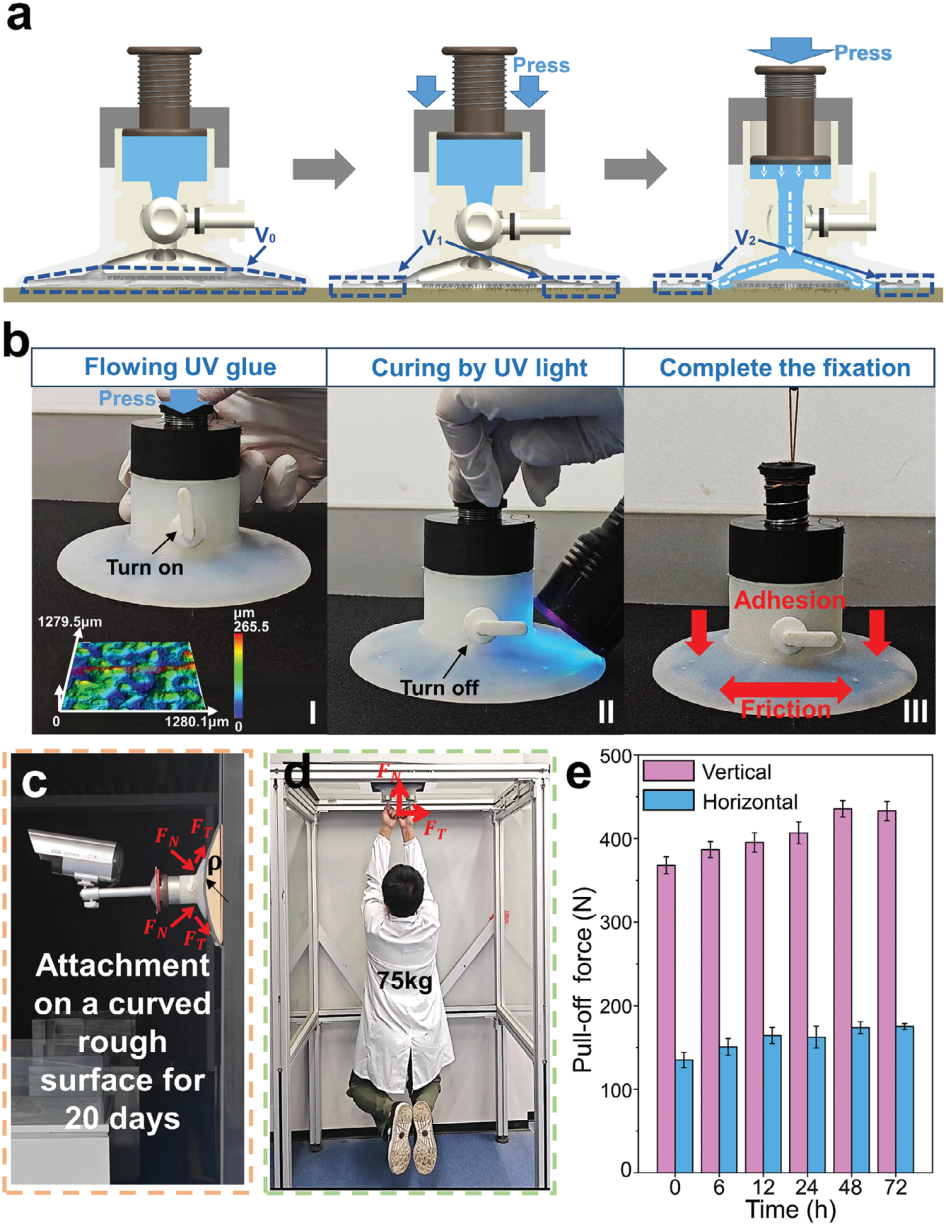
Practical applications of the bionic fixation device. a) The process of discharging air from the device. b) Process of device placement. c) Fixation of a surveillance camera using the device on a curved rough surface for 20 days. d) Carrying an adult weighing 75 kg on a rough surface using two devices. e) The pull‐off force‐time curve for the device.

The successful attachment of the bionic fixation device is demonstrated to different rough surfaces (multi‐material surfaces, curved rough surfaces, and horizontal rough surfaces) under a variety of working conditions. **Figure** **6**a demonstrates the process of discharging air from the device. When the device is just placed on the contact surface, there is air of volume *V*_0_ between device and surface. When the device is pressed into contact with the surface (before UV glue flows down), a part of the air is discharged, leaving only air of volume *V*_1_. In this state, the device can adhere to smooth surfaces without UV glue (as shown in Figure 2b,c). After that, pressing the push rod to squeeze UV glue into the suction disc guide channels discharges the remaining air with the volume of *V*_2_. The volume *V*_2_ will gradually decrease with the flow of UV glue, which is an important reason for the device to be able to adhere to rough surfaces. Figure 6b shows the process of a device fixed on a rough surface (*S*_a_ ≈ 28 µm). The general steps are placing the device, opening the valve to make UV glue flow, using UV light to irradiate the curing, and completing the fixation (Video S5, Supporting Information). Figure 6c shows a surveillance camera fixed on a curved rough wall surface (*S*_a_ ≈ 28 µm) for 20 days by a bionic fixation device, demonstrating the device's ability to achieve long‐term fixation on curved, rough surfaces. Figure 6d shows that an adult male weighing 75 kg can be supported by two bionic fixation devices (Video S6, Supporting Information) even if they are attached to a rough surface (*S*_a_ ≈ 28 µm). Figure 6e illustrates the trend of the device's desorption force over time. With the time increasing, the pull‐off force of the device increases and finally stabilizes. The reason for this is that the adhesive strength and hardness of the cured UV glue increases with time, which enhances the chemical bonding and mechanical locking with the suction disc guide channels, so the pull‐off force also increases. When the cured UV glue reaches its maximum strength at 48 h, the pull‐off force of the device remains stable. The device also achieves excellent attachment even in moist environments (Figure S12, Supporting Information). The above applications demonstrate the strong adaptability and mechanical properties of the bionic fixation device to a wide range of rough surfaces. Since the device is a multi‐mechanism fixation, different fixing mechanism can be selected depending on the application environment and stress conditions (Figure S15, Supporting Information).

## 3 Conclusion

In this study, a bionic fixation device consisting of a synergistic combination of three mechanisms is proposed: negative pressure adsorption, mechanical locking, and chemical bonding. The device is based on the end‐expanded suction disc structure equipped with a gradient guide channel at the bottom, a microneedles friction‐enhancing unit, and fast‐curable UV glue. This study prepared several bionic fixation devices with different structures and experimentally analyzed the effect of structural inhibition on their attachment ability. The complete device has the best attachment ability due to the cooperative multiple mechanisms, which results in the maximum detachment work (5.7 and 5.5 J) and pull‐off force (377 and 175 N) on different rough surfaces, separately detached vertically and horizontally by pulling. The pull‐off force‐time curves of the devices with different structures in vertical and horizontal pulling show the decisive role of the negative pressure adsorption mechanism in the pull‐off force. The internal vacuum of the device determines the upper limit of the pull‐off force, and the mechanical locking and chemical bonding mechanisms slow down the pull‐off force decrease and increase the time of desorption. This multi‐mechanism strategy of increasing energy consumption allows the device to better resist external loads. Experiments with microneedles backing demonstrated the adaptability of soft‐backed microneedles to rough surfaces. In addition, a comparison of UV glue flow velocity in several suction discs with different angles of guide channels demonstrated the presence of Laplace pressure driving UV glue flow in the guide channels, of those, an angle of 4° proved to be the most efficient for transporting UV glue. Finally, the application feasibility of the bionic fixation device was demonstrated in combination with a surveillance camera laid on rough surfaces and demonstrated its strong adhesion ability by two devices carrying a 75 kg adult on a rough surface. The pull‐off force‐time curve for the device and fixation of a surveillance camera using the device on a curved rough surface for 20 days demonstrated that the device can realize long‐term fixation.

## 4 Experimental Section

### Pull‐Off Force Measurements of Devices

To study the cooperative benefits of multi‐mechanism, four devices with different structures were fabricated and tested. The detachment curves were measured for devices on sandpapers with different grain sizes (Figure S6, Supporting Information), such as 80 mesh (grain size ≈ 178 µm), 120 mesh (grain size ≈ 124 µm), 220 mesh (grain size ≈ 68 µm), 400 mesh (grain size ≈ 38 µm), 1500 mesh (grain size ≈ 11 µm), 5000 mesh (grain size ≈ 3 µm). These measurements were taken in two directions using a Jingzhuo universal testing machine (Yangzhou Jingzhuo Testing Machine Factory, China) with 500 N Transcell sensor and a homemade horizontal force measuring platform with 500 N Transcell sensor respectively (Figures S3 and S4, Supporting information). To ensure the attachment of the devices, a preload of 10 N for 10 s was applied to devices before the initiation of each experiment. After releasing UV glue to complete the curing, the devices were detached at a certain speed (8 mm s^−1^). The experimental curves of the pull‐off force‐time were measured by connecting to a computer to calculate the detachment work. The detachment processes of devices were recorded (Videos S1–S3, Supporting Information). All measurements were repeated at least five times and averaged values were displayed.

### Friction Measurements of Microneedles

The same homemade horizontal force platform was used for these measurements. As shown in Figure 4d, the measurements were performed by fitting the weights to the top of the device, using a non‐stretchable rope over the lower part of the device, pulling the device, and recording the maximum friction that a bionic fixation device with two types of backing microneedles could withstand after being pulled horizontally from rest to movement. All measurements were repeated at least five times and averaged values were displayed.

### Full Flow Time Measurements of UV Glue in Different Suction Discs

To investigate the ability of the suction disc's gradient guide channels, differences in the self‐driving ability of the five suction discs were demonstrated by the time it takes for UV glue to flow full of the guide channels. After applying preload to the device to make good contact with the rough surface, the valve of the ball valve was opened and the UV glue flowed down. The time and dynamic process of UV glue to flow full of the suction disc guide channels were recorded (Video S4, Supporting Information).

## Conflict of Interest

The authors declare no conflict of interest.

## Supporting information



Supporting Information

Supplemental Video 1

Supplemental Video 2

Supplemental Video 3

Supplemental Video 4

Supplemental Video 5

Supplemental Video 6

## Data Availability

The data that support the findings of this study are available from the corresponding author upon reasonable request.
