# Supplementary material for: Multi‐Mechanism Collaborative Bionic Fixation Technique Between a Wide Range of Solid Interfaces
Source: Adv Sci (Weinh). 2024 Nov 26;12(3):2409507. doi: 10.1002/advs.202409507 (PMC11744652; doi:10.1002/advs.202409507)
Supplement: Supplementary file 1 — Supporting Information [file ADVS-12-2409507-s007.docx]

# Supporting Information

# Multi-mechanism collaborative bionic fixation technique between a wide range of solid interfaces

*Shixun Fu^1^, Jun Sun^2^, Zhiyong Hu^3^, Yongjin Zhao^4^, Tianchang Yao^4^, Xipeng Wang^1^, Yuanming Ji^1^, Kai Deng^1^, Keju Ji^1^**

***
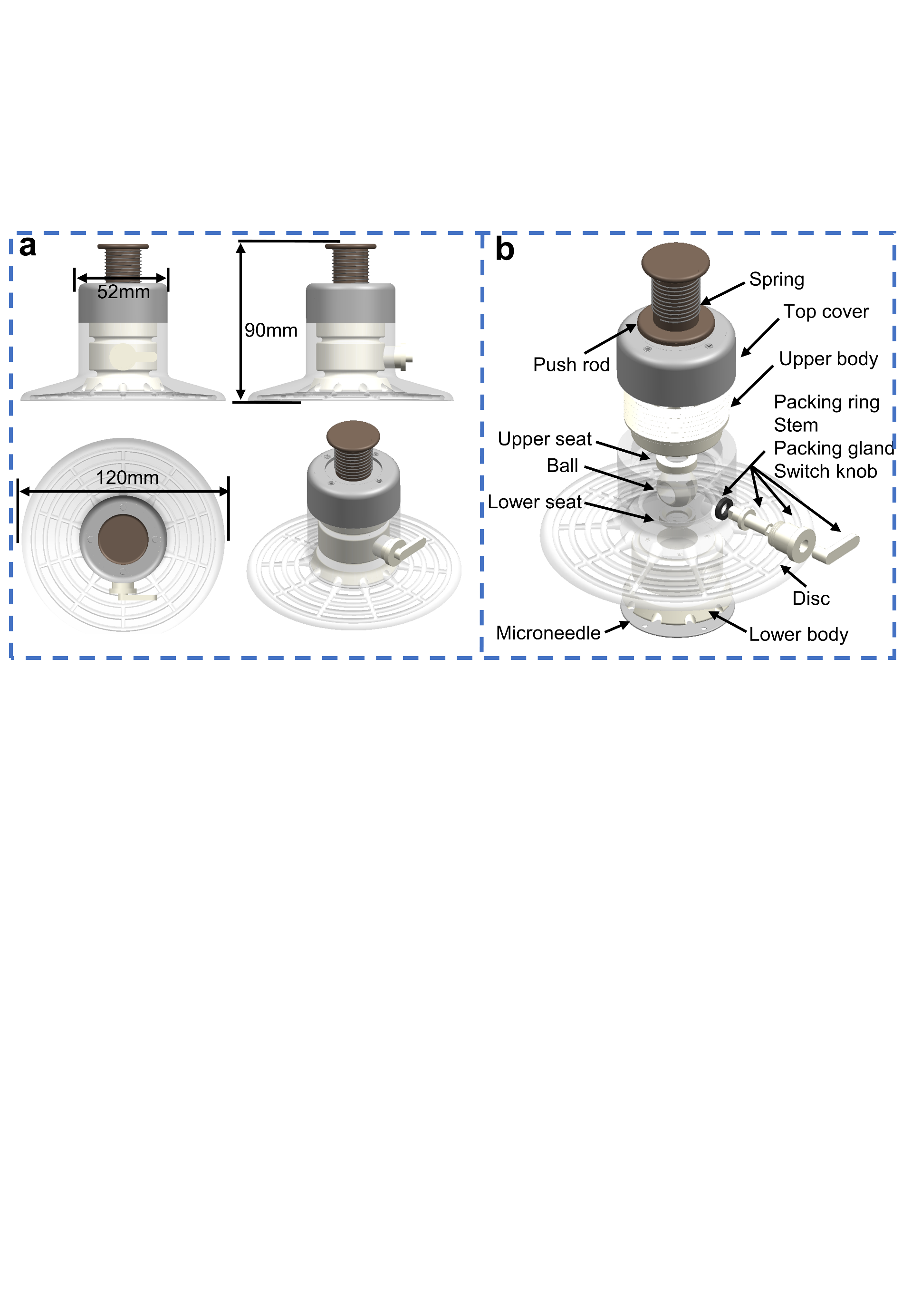
*Figure S1.** a) Main sizes and b) parts of the bionic fixation device.

**
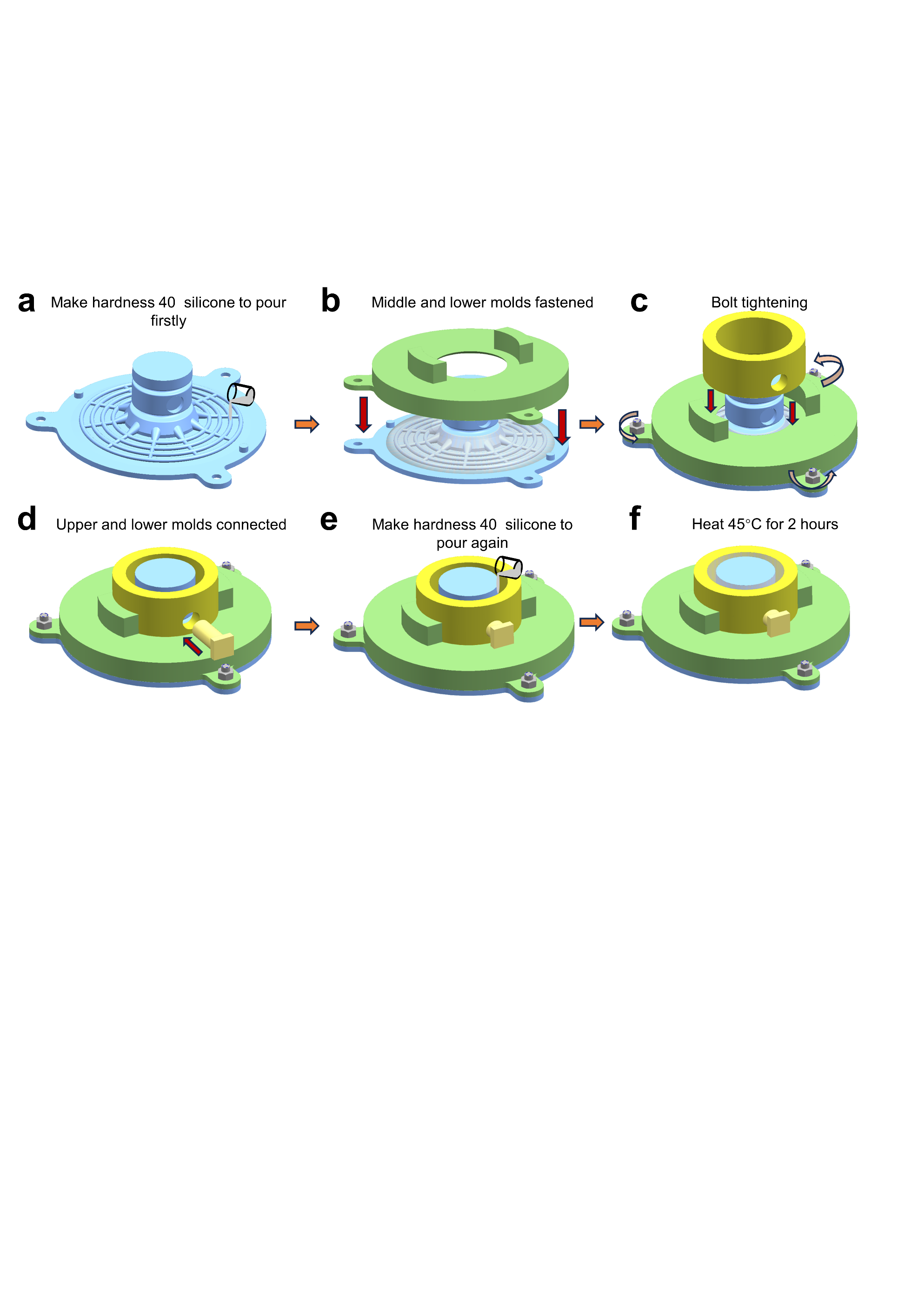
Figure S2.** Manufacturing process of the suction disc. a) Prepare a 40 hardness silicone, vacuum it and pour it into the lower mold overflowing the guide groove. b) Through the pin on the lower mold and hole on the middle mold, fasten the middle and lower molds accordingly. c) Bolt down the middle and lower molds and place the upper mold. d) Through the pin, fasten the upper and lower molds accordingly and use this method to create the hole in the upper part of the suction disc. e) Prepare a 40 hardness silicone, vacuum it and Use it to fill the remaining space in the mold. f) Heat in an oven at 45 ℃ for 2 hours.

**
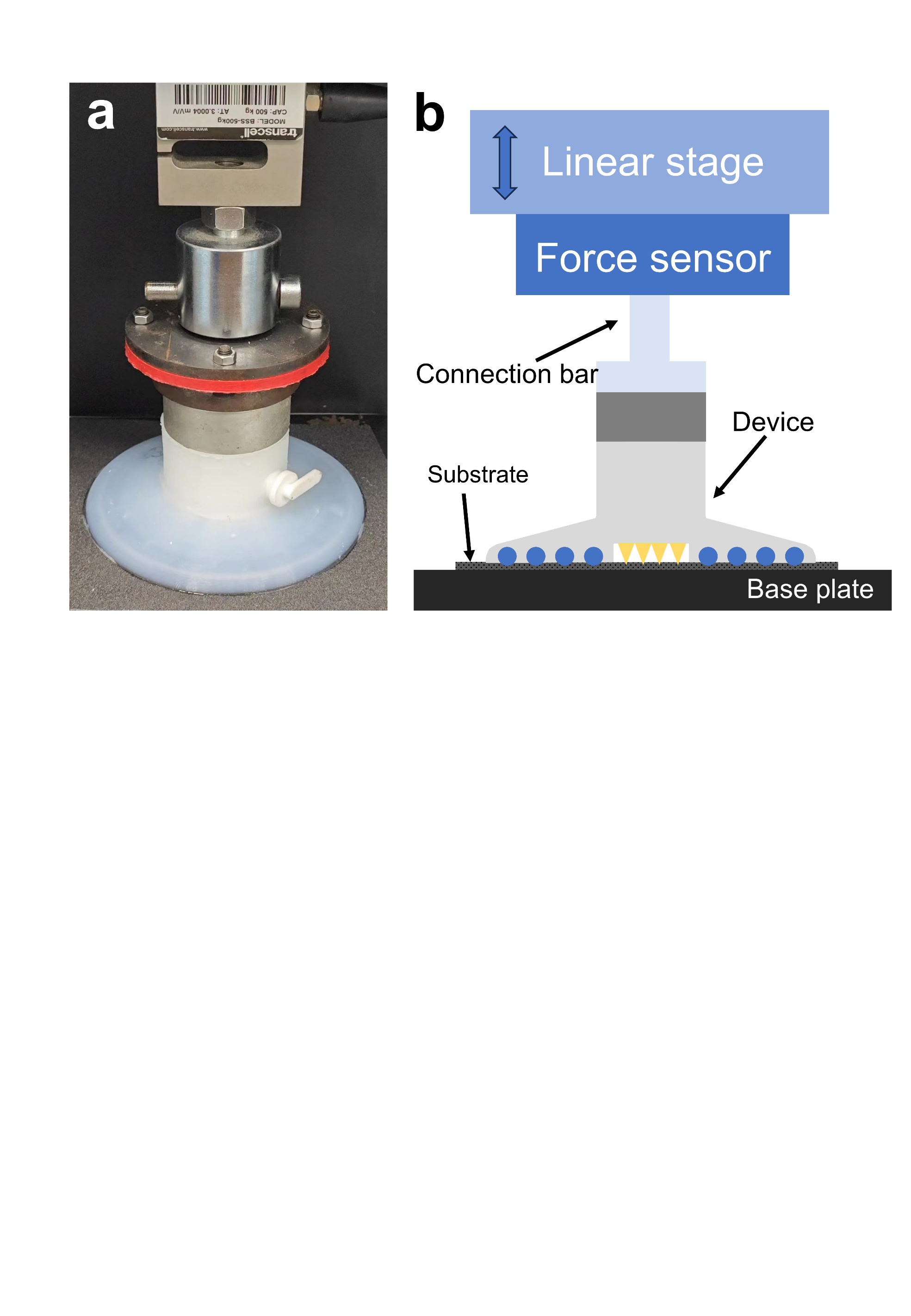
Figure S3.** a) Experiments on the pull-off force after vertical pulling is measured using a universal stretching machine, and b) schematic of the experimental setup.


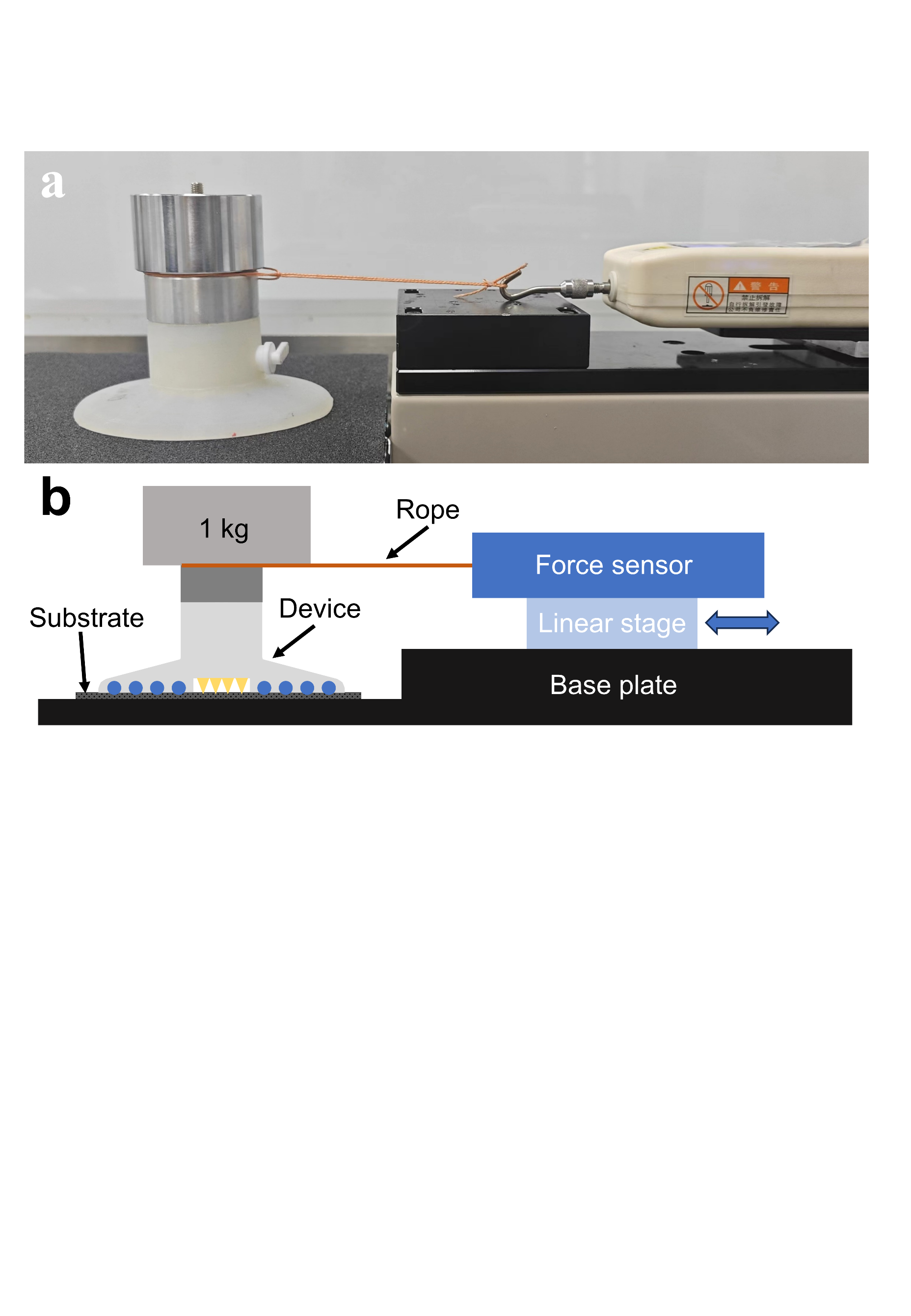


**Figure S4.** a) Experiments on the pull-off force after horizontal pulling is measured using a horizontal force measuring platform, and b) schematic of the experimental setup.

**
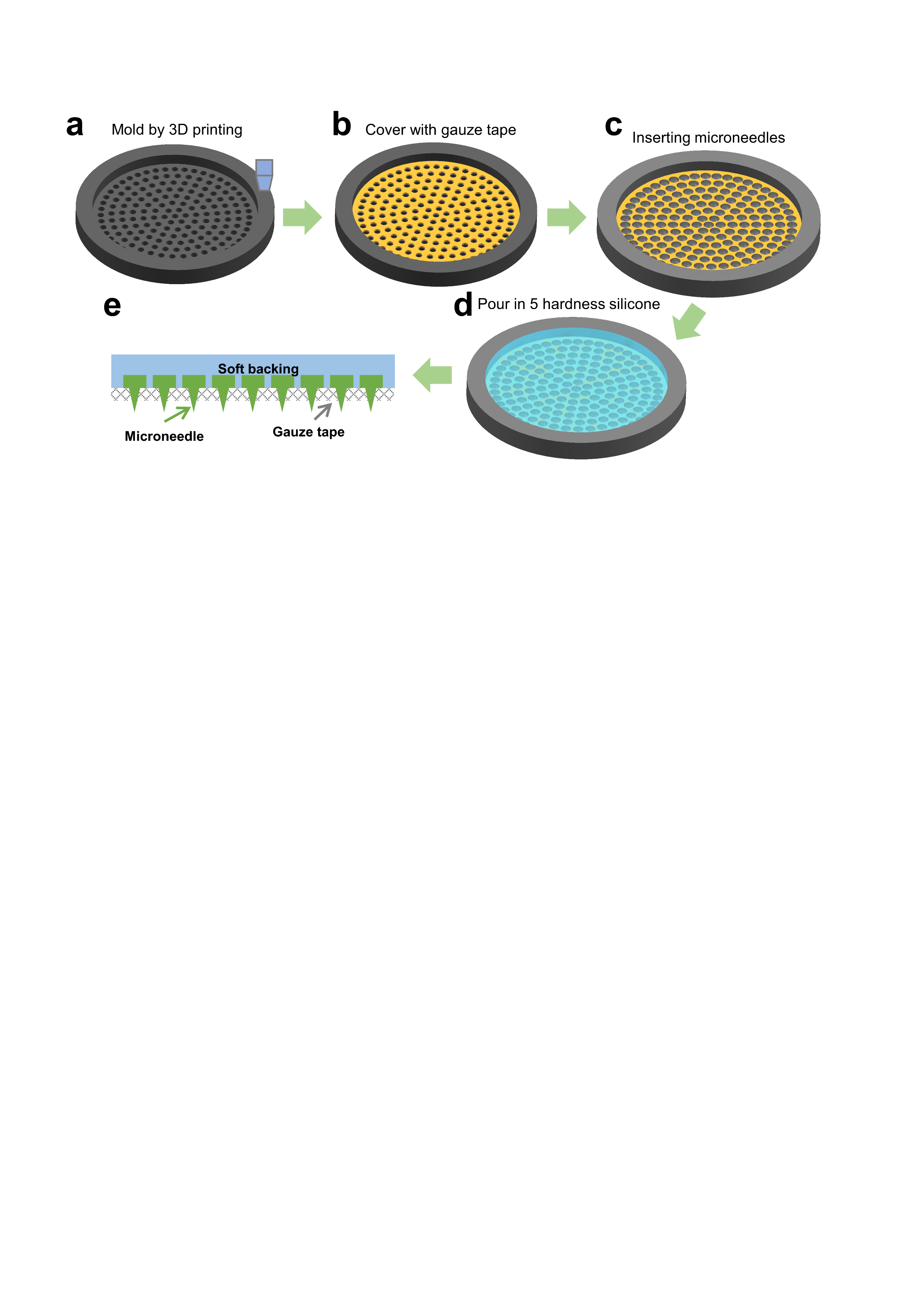
Figure S5.** Manufacturing process of the soft-backed microneedles. a) Using a 3D printer to print a mold with a circular array of through holes at the bottom. b) Cover the mold with machine-processed gauze tape, with holes corresponding to the holes. c) Embed the microneedle into the corresponding hole to form microneedle arrays. d) Prepare silicone of hardness 5 and fill to the top of the mold, then put it in the oven at 45 °C for 1 hour. e) A soft-backed microneedles with a silicone top layer, a gauze tape middle layer, and a microneedles bottom layer is obtained.


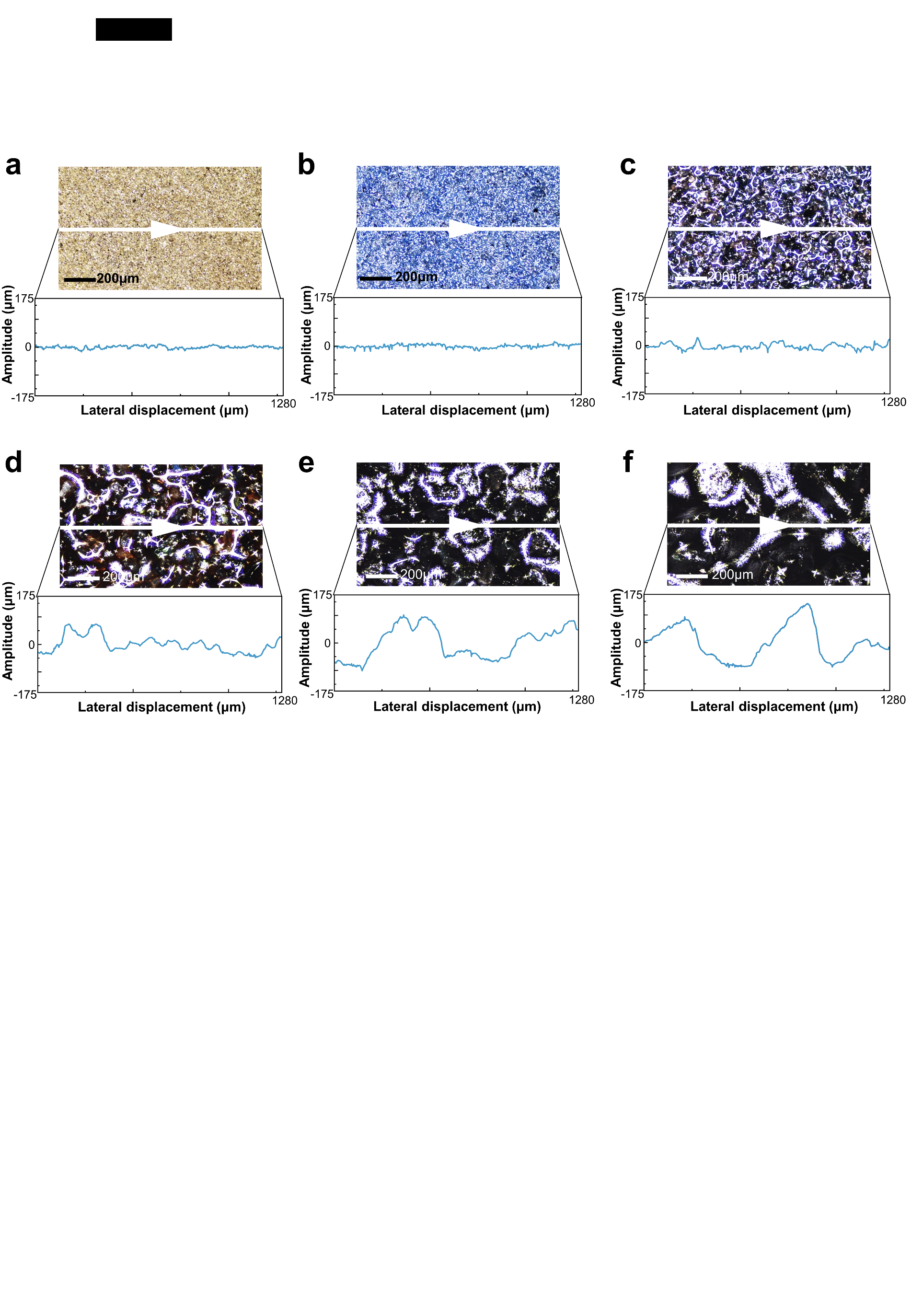


**Figure S6**. Surface images and cross-sectional profiles of sandpaper with different grain sizes. a) P5000, grain size = 3 μm. b) P1500, grain size = 11 μm. c) P400, grain size = 38 μm. d) P220, grain size = 68 μm. e) P120, grain size = 124 μm. f) P80, grain size = 178 μm.

**
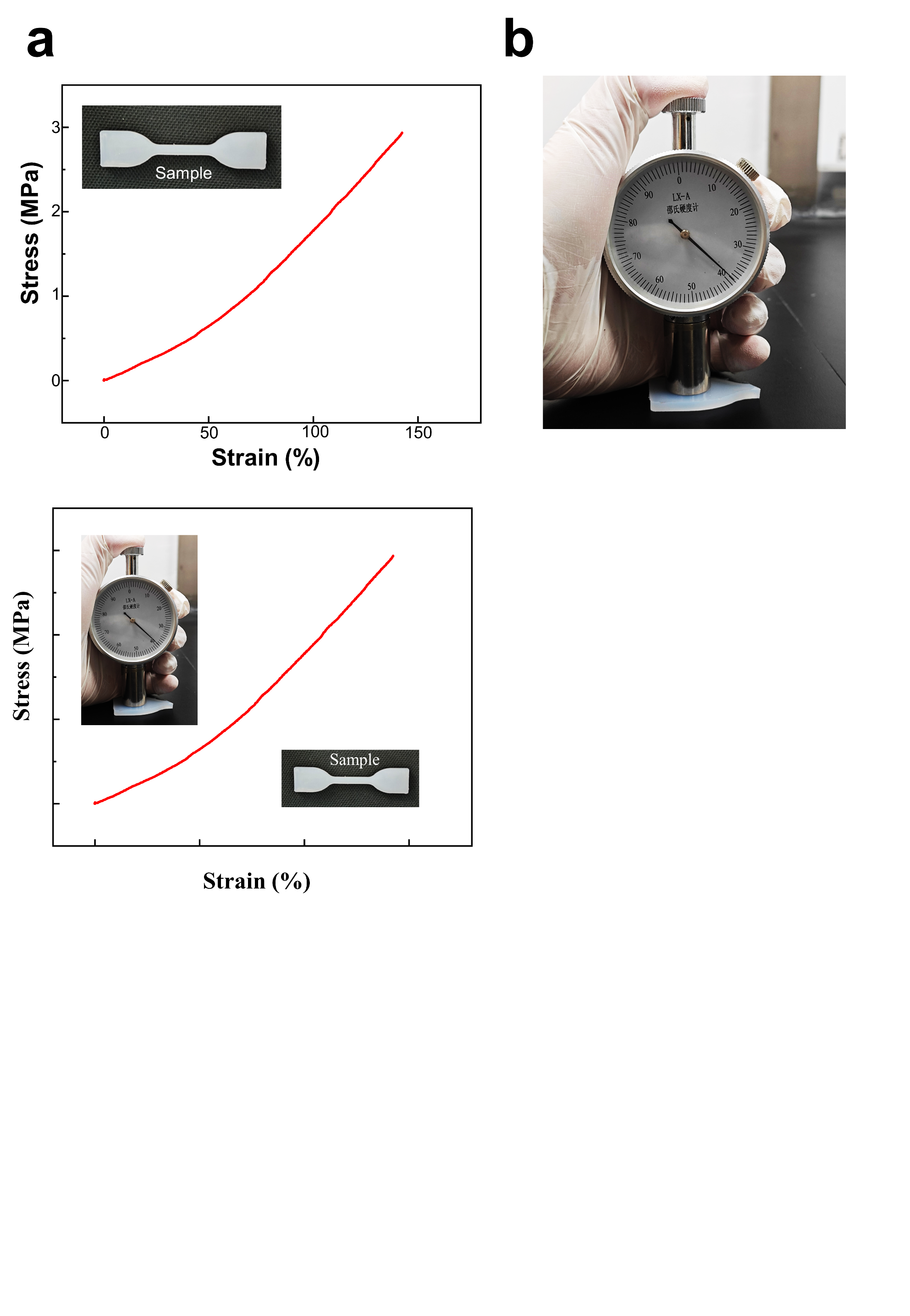
**

**Figure S7**. Suction disc material performance. a) Stress-strain curve of suction disc material. b) Hardness of the suction disc.

Suction disc material has excellent deformation ability. The tensile stress-strain curve of the material (Figure S7) is tested using the testing machine (JZL-D, JINGZHUO TESTING INSTRUMENT), and observed Young’s modulus was 0.901 MPa. Figure S7b shows that the Shore hardness of the material is 40A. This **silicone rubber was purchased from Beijing Sanjing Xinde Technology Co., Ltd.**


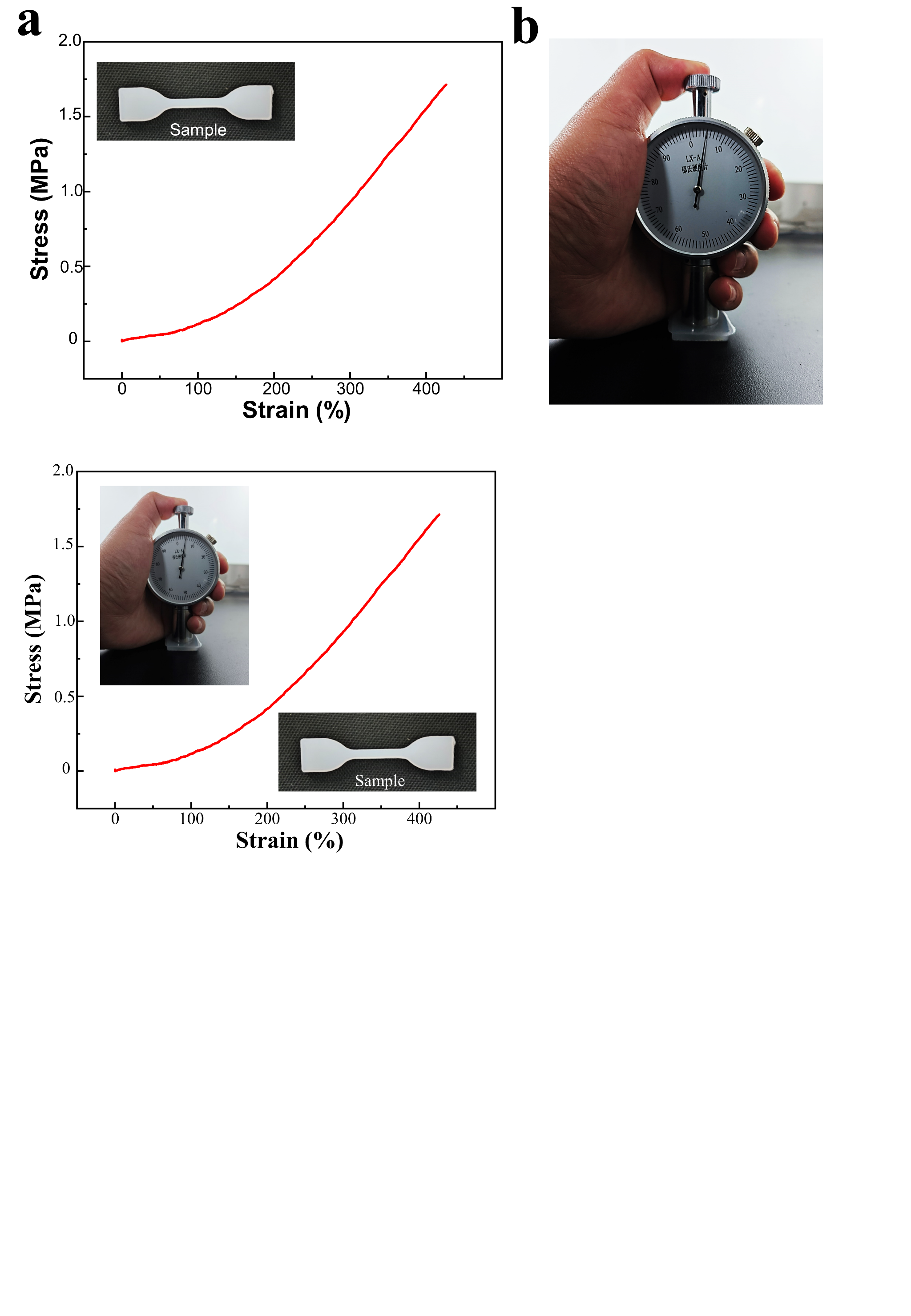
 **Figure S8**. Soft backing material performance. a) Stress-strain curve of soft backing. b) Hardness of the soft backing.

The tensile stress-strain curve of the material (Figure S8) is tested using the testing machine (JZL-D, JINGZHUO TESTING INSTRUMENT), and observed Young’s modulus was 0.0854 MPa. Figure S8b shows that the Shore hardness of the material is 5A. This **silicone rubber was purchased from Beijing Sanjing Xinde Technology Co., Ltd.**

**
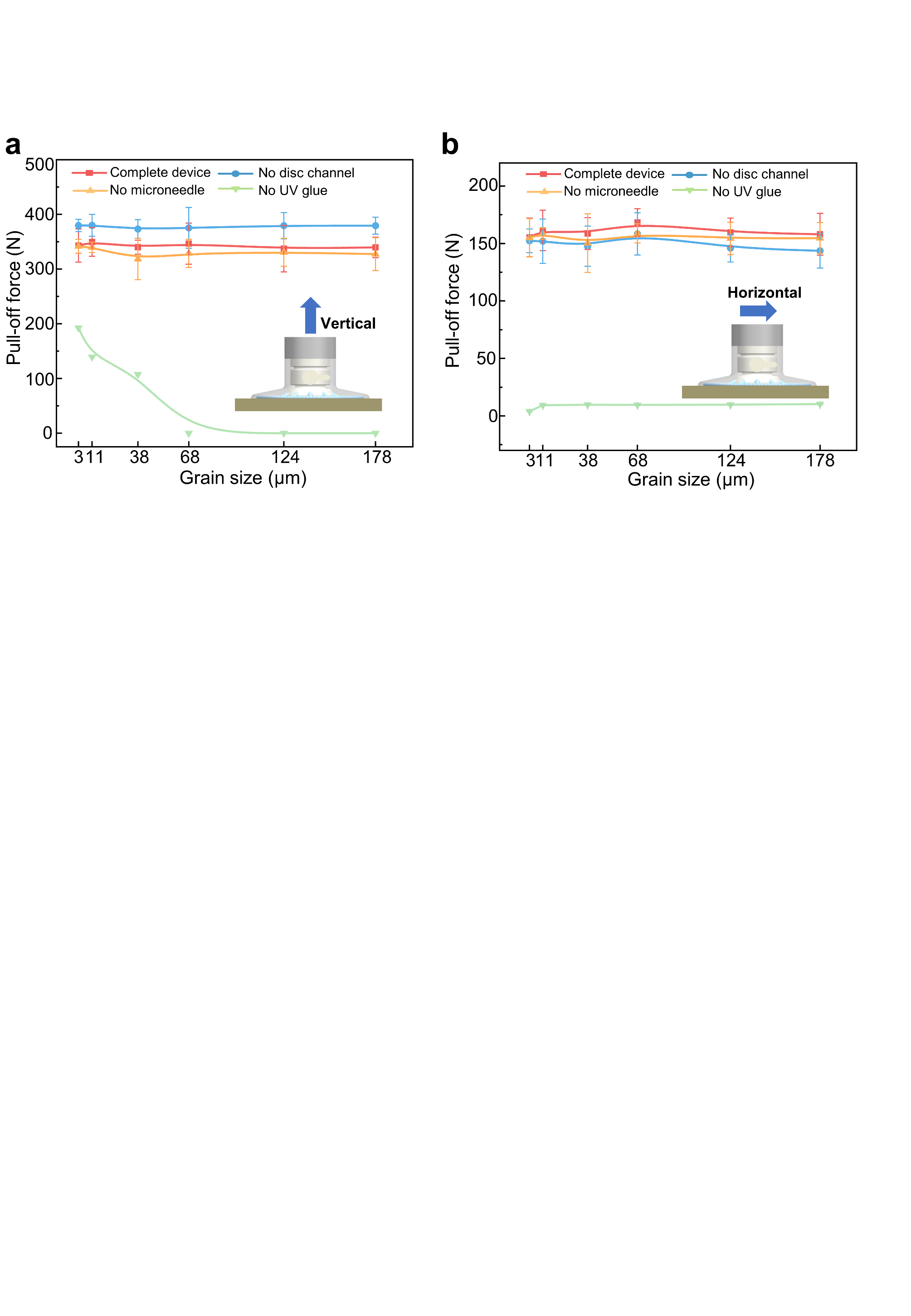
**

**Figure S9.** Measurements of pull-off force for different devices and different conditions. a) vertical, b) horizontal.

**
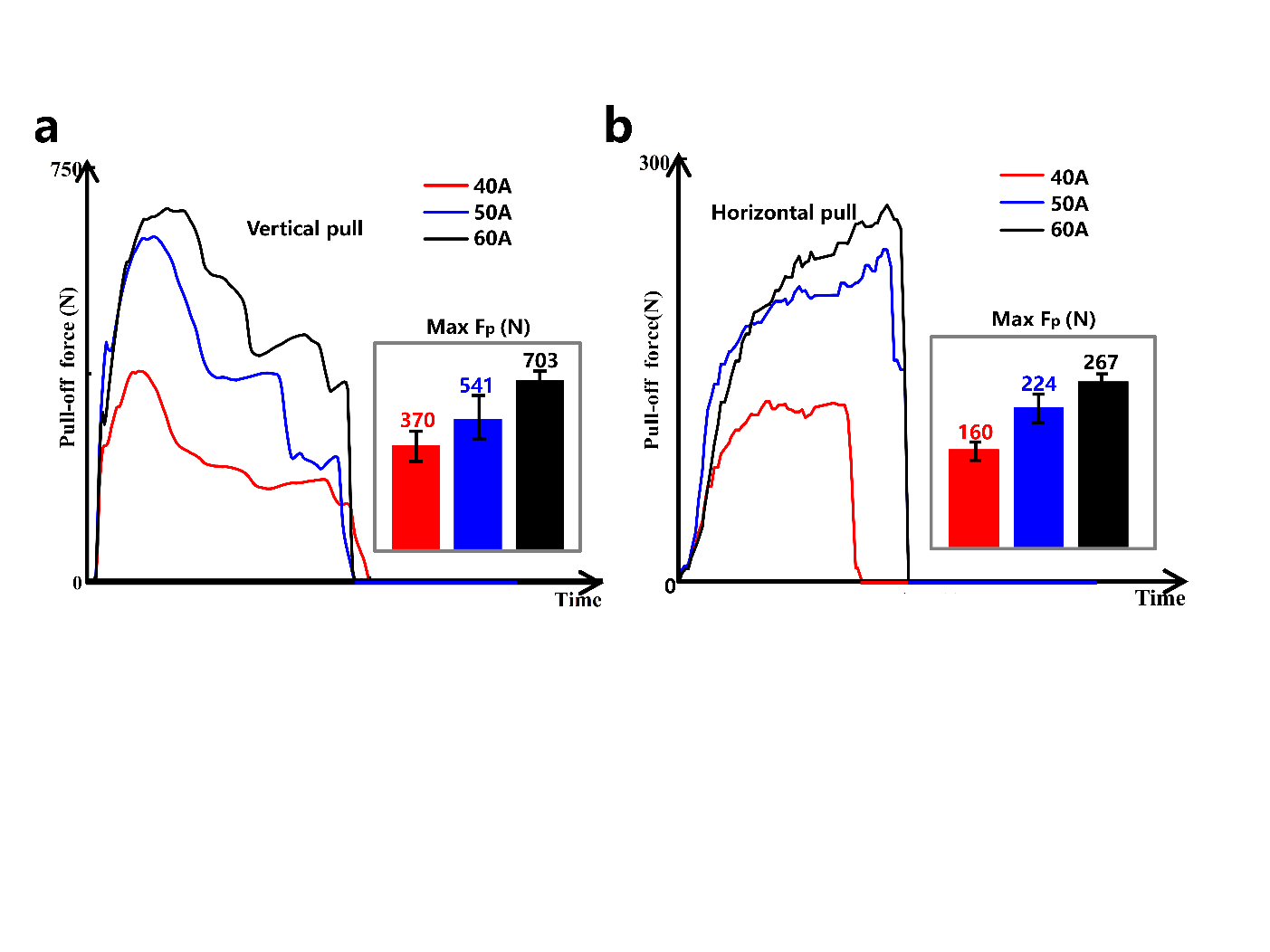
**

**Figure S10**. Pull-off force-time curves for the bionic fixation device equipped with suction discs of different harnesses (grain size ≈ 124 μm). a) Vertical pull. b) Horizontal pull.

Normally, as the hardness of the suction disc increases, the suction disc becomes less adapted to rough surfaces, which leads to a sharp decrease in the pull-off force, but the curves do not show this trend, instead, the pull-off force tends to increase as can be seen from Figure S10. The reason that the pull-off force is not weakened is that the presence of UV glue ensures that no matter how poorly the suction disc adapts to rough surfaces, the flow of UV glue fills the gap between them and achieves a sealing effect.


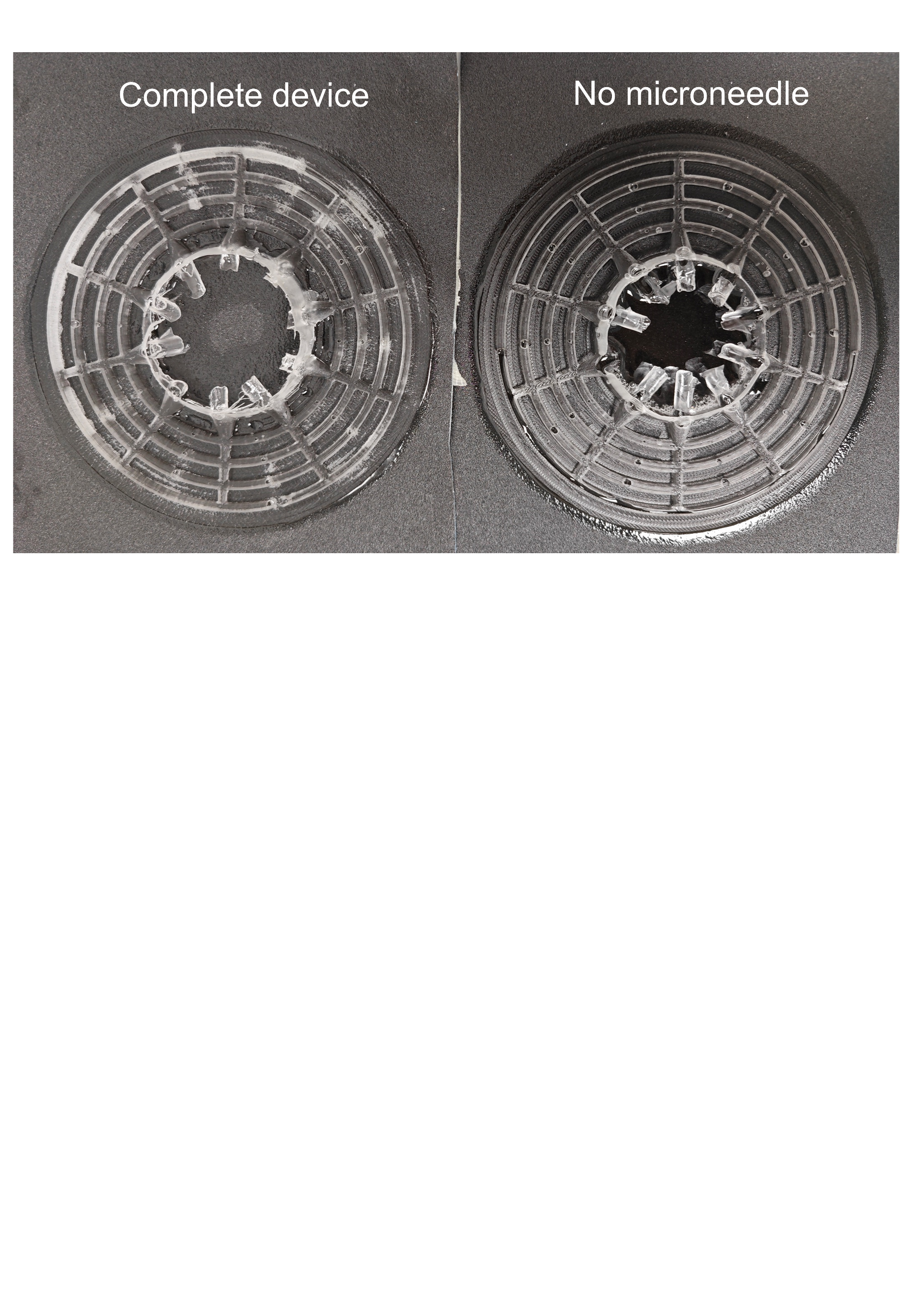


**Figure S11.** Comparison of the UV glue residue on the bottom of the complete device and the no microneedle device.


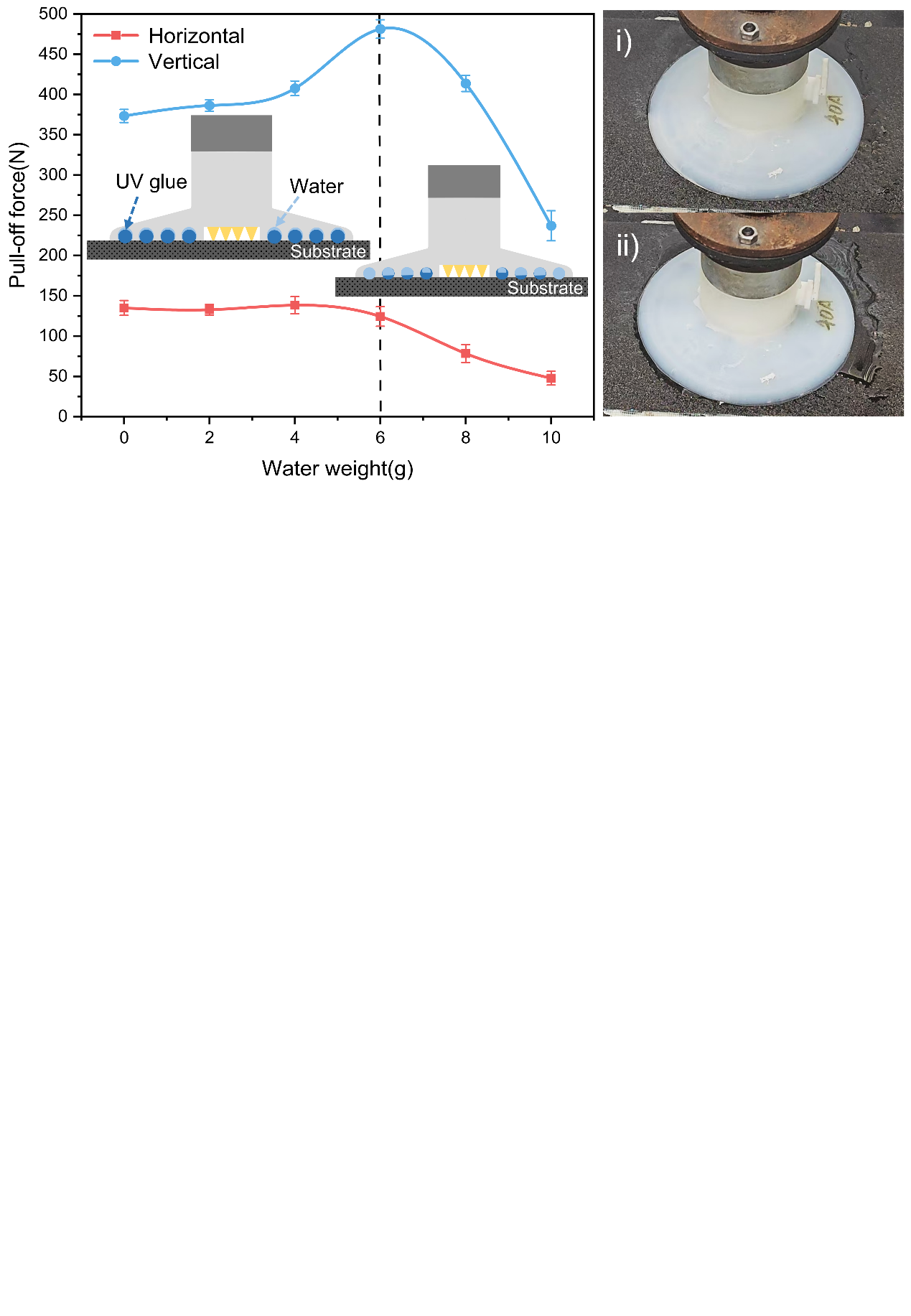


**Figure S12.** Pull-off force of the device in the moist environments.

As shown in Figure S12, as the moisture level of the substrate surface increases, the trend of the pull-off force in both the vertical and horizontal directions increases and then decreases. When the device is placed on a wet substrate, water on the surface will remain in the substrate's crevices as well as in the suction disc's guide channels. UV glue is not soluble in water. As the UV glue flows into the suction disc's guide channels, the UV glue gradually discharges the water between the suction disc and the substrate (Figure 2-3). When the water on the substrate surface is very little, with the UV glue flow full of suction disc guide channels will discharge water, curing UV glue with UV lamps to generate heat will also evaporate part of the water. At this time, the device's fixation state is similar to the state on the dry surface, so the pull-off force does not change much, but also shows that the water does not affect the curing of UV glue. When the water on the substrate surface increases, the UV glue curing process evaporation part of the water can not be discharged to increase the volume of the cavity. At the same time there is also a small amount of water forming a water film between the device and the cured UV glue to enhance the friction and adhesion between the cured UV glue and device. So the pull-off force is greater compared with the dry environment. When too much water on the substrate surface, the device is placed on the substrate surface, the suction disc guide channels are mostly infiltrated in the water, UV glue flowing into the guide channels can still discharge water, but the amount of water present in the suction disc guide channels is too much to take up the space where the UV glue can flow. The UV glue can only flow in the innermost two rings of the guide channels. The UV glue can still be cured normally, but can not be sufficiently full of the guide channels of the suction disc, so the device has a reduced pull-off force.


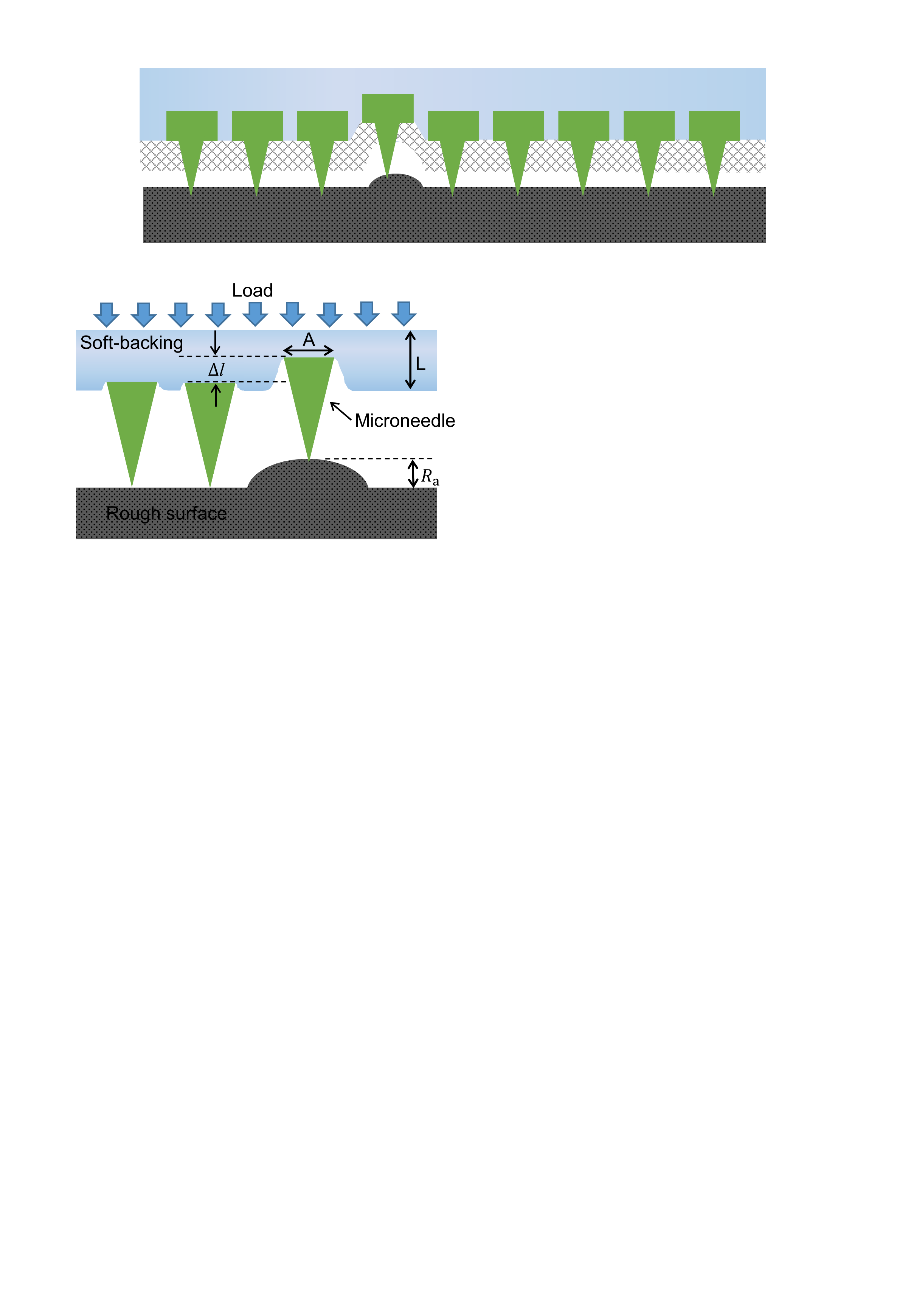


**Figure S13.** Force analysis of soft-backed microneedles

As shown in Figure S13, when a load $F_{load}$ is applied to the device, each microneedle is subjected to a force ${{F_{n}\text{ }\text{=F}}_{\text{load}\text{ }}}/{\text{ }\text{n}}$, where *n* is the number of microneedles. When a single microneedle is pressurized into contact with a rough surface, the backing on it deforms $\text{∆h}\text{ }\text{=}\text{ }{\text{F}_{\text{load}\text{ }}\text{H}}/{\text{ }\text{nAE}}$. As the microneedle gradually reaches the highest point of the rough surface bump, the force $F_{n}\text{ }\text{=}\text{ }{\text{F}_{\text{load}}}/\text{n}\text{+}\text{nAE∆l}/\text{H}$ increases with increasing backing deformation. So the static friction provided by all soft-backed microneedles is $\text{F}_{\text{f}}\text{ }\text{= μn}{\int F_{n}d\Delta h}/{R_{a}}\text{ = μ}\left( F_{load}\text{+}{\text{n}^{\text{2}}\text{AE}\text{R}_{\text{a}}}/\text{ 2H} \right)$, where $\mu$ is the coefficient of static friction between the microneedle material and the rough surface; *A* is the cross-sectional area of the end of the microneedle; $\text{R}_{\text{a}}$ is the surface roughness; *E* is the elastic modulus of the backing; *H* is the thickness of the backing. From the above equations, it can be seen that the friction force is $\text{F}_{\text{f}}$ affected by the elastic modulus of the backing and the number of microneedles. As the backing elastic modulus increases, the friction increases. However, there is a maximum value of the modulus of the backing $E_{max}\text{ }\text{=}{{\text{ }\text{F}}_{\text{load}}\text{H}}/{\text{nA}\text{R}_{\text{a}}}$. Since the modulus of elasticity of the backing is too large, the deformation under pressure is too small, and it will be similar to the case of hard-backed microneedles, which is difficult to adapt to rough surfaces. An increase in the number of microneedles also increases friction, but there is also a maximum in the number $n_{max}\text{ }\text{=}{\text{ }\text{AED}}/{\text{(υ}\text{F}_{\text{load}}\text{+}{\text{(}{\text{A}^{\text{3}}\text{E}^{\text{2}}}/\text{π}\text{)}}^{\frac{\text{1}}{\text{2}}}}\text{)}$, where *D* is the diameter of the backing; υ is the Poisson's ratio of the backing.


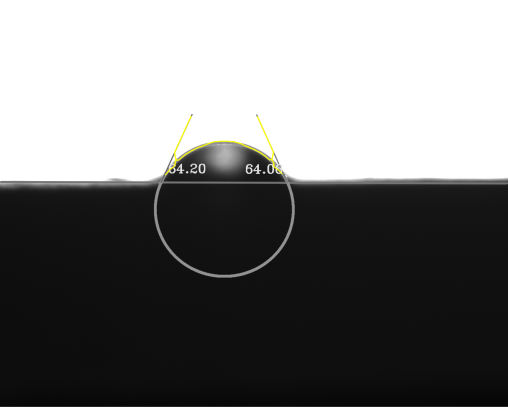


**Figure S14.** Contact angle of UV glue on the surface of silicone

The contact angle of UV glue on the surface of silicone was measured by using contact angle test equipment (DSA-X, KEJING). The measured contact angle is 64.1°.


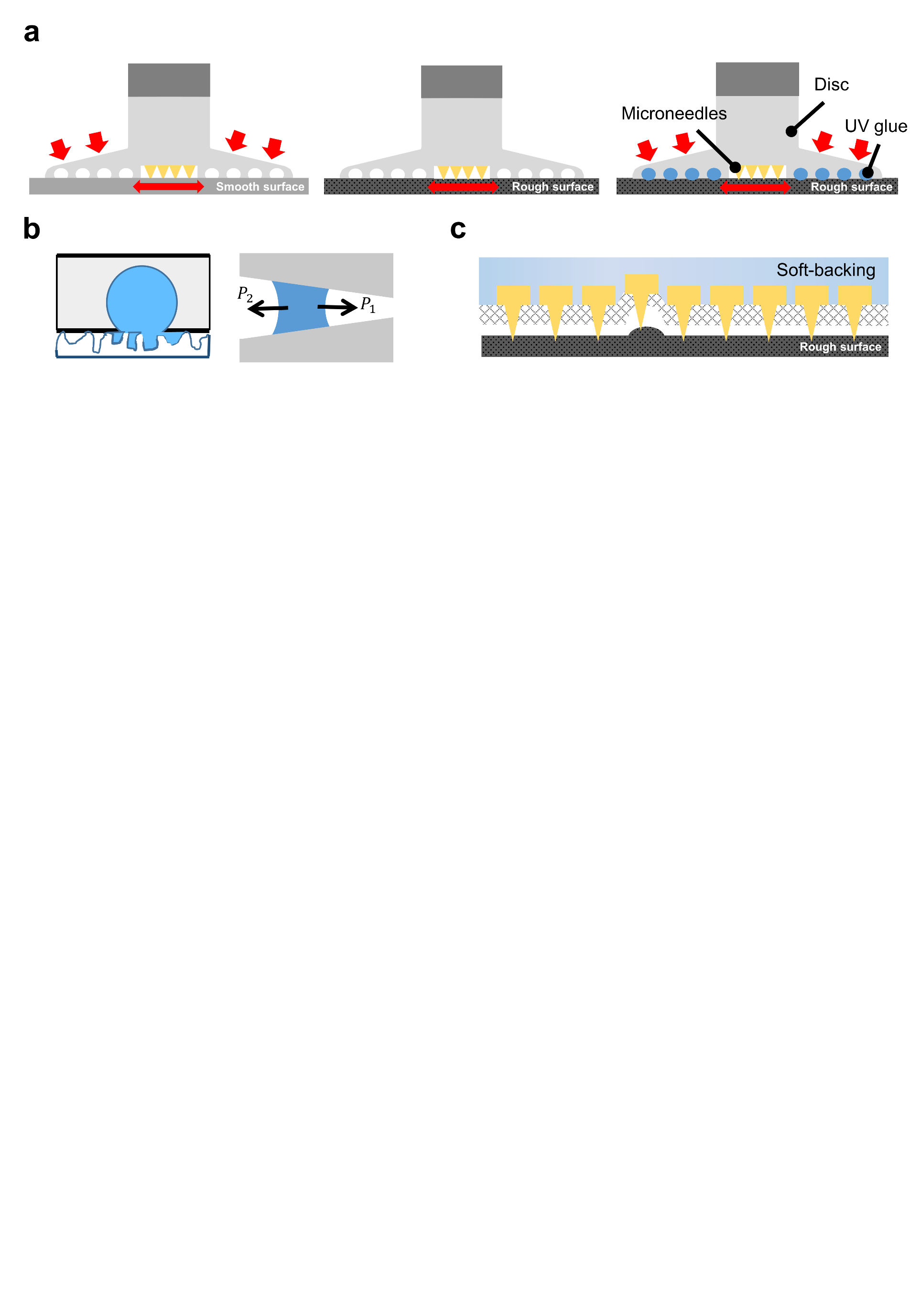


**Figure S15.** Different fixation options for different rough surfaces


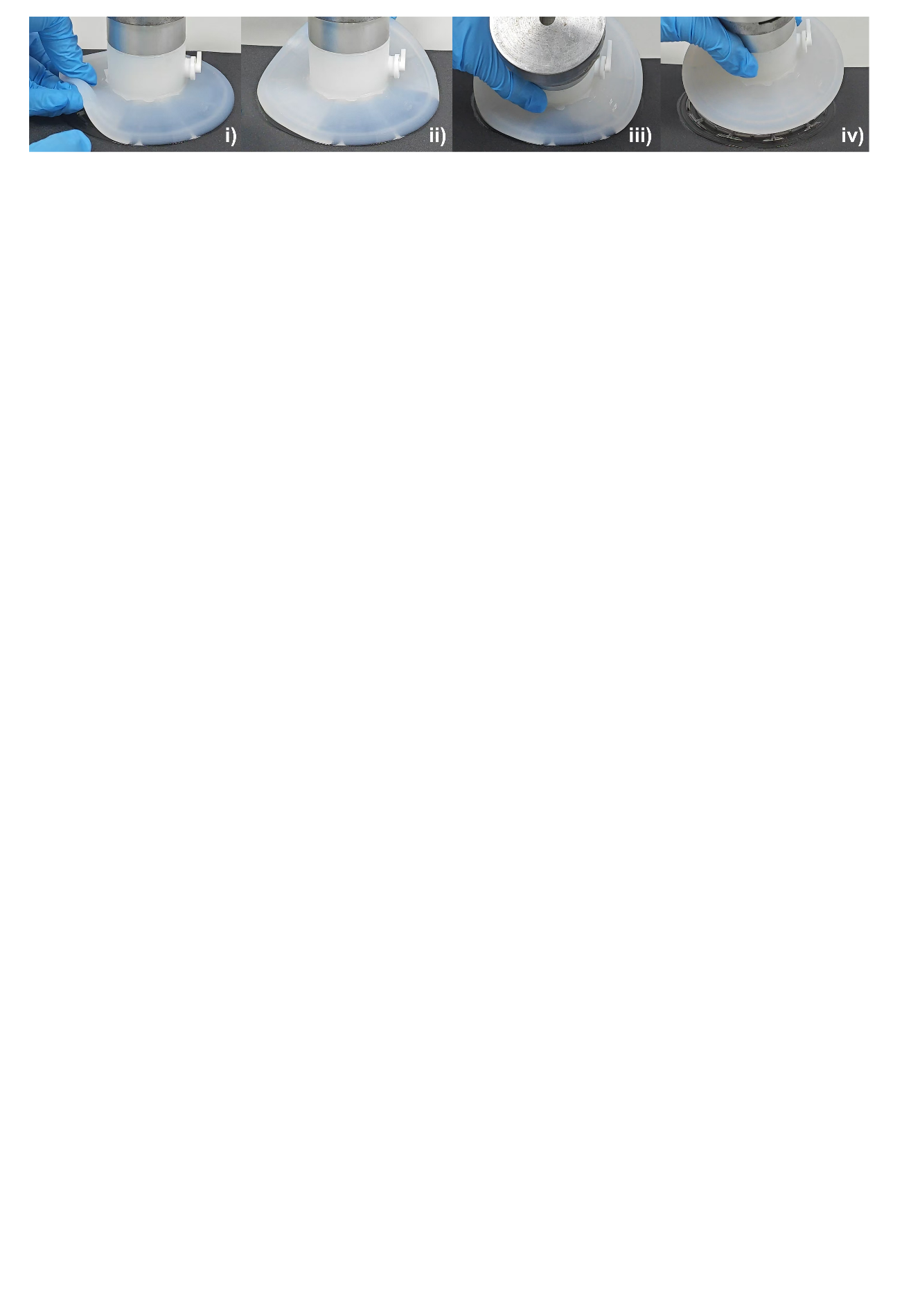


**Figure S16.** Desorption of the bionic fixation device.


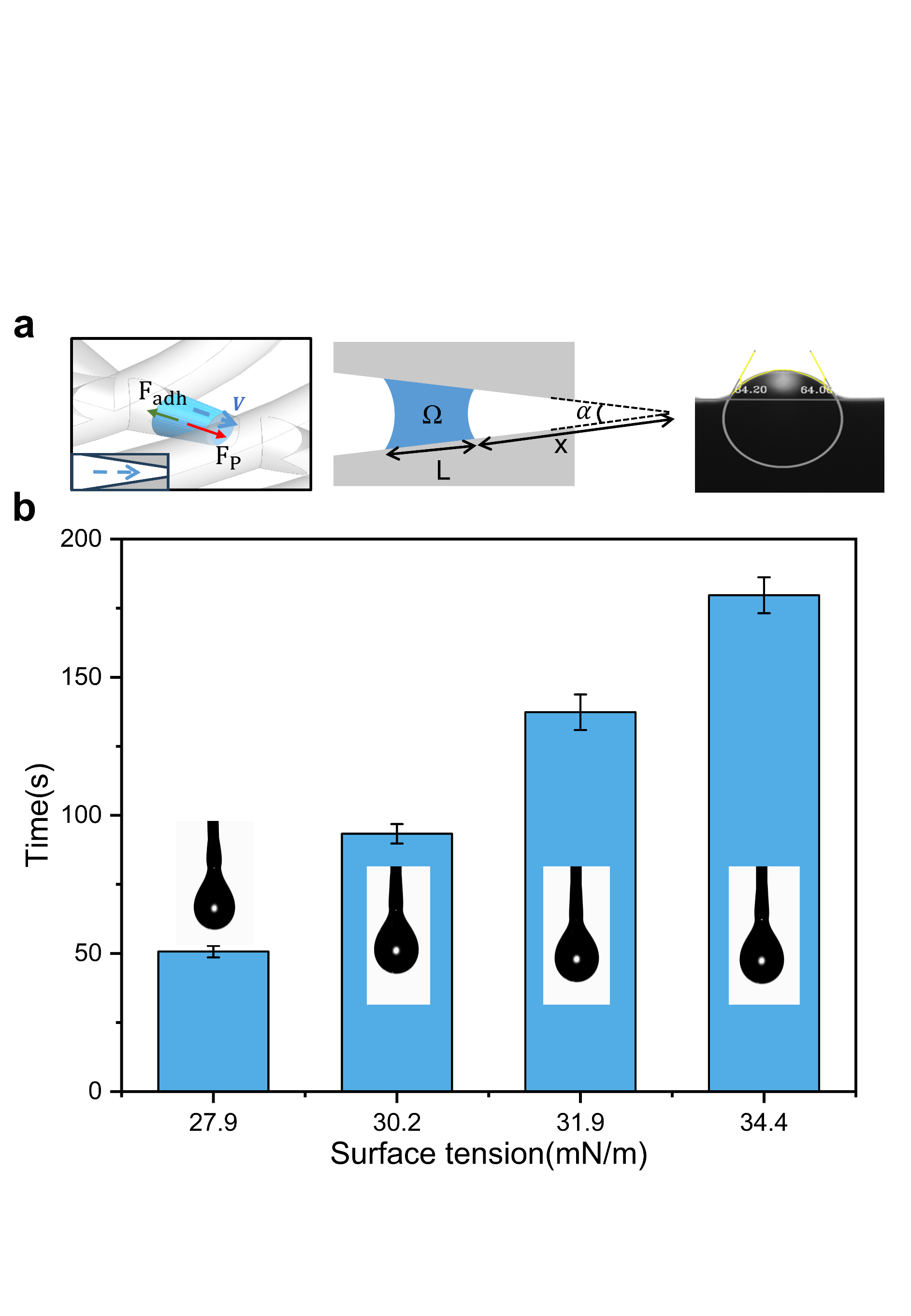


**Figure S17.** Time for UV glue with different surface tension to flow full of suction disc guide channels

We measured the surface tension of the UV glue using a contact angle measurement device (DSA-X, KEJING) selecting the pendant drop method. As shown in Figure S17 the time increased with increasing surface tension $\gamma$. The reason can be explained by combined force $F=F_{p}-F_{adh}=\gamma({8\Omega^{2}}/{\pi\alpha^{2}x^{5}}\cos\theta-\alpha x\Delta\cos\theta)$, where $\Omega$ is the volume of the droplet; *x* is the distance from droplet to the top; $\alpha$ is the angle of the suction disc guide channel; $\theta$ is the contact angle between UV glue and suction disc. For partially wetted liquids like UV glue (contact angle $\theta$ is 64°), the Laplace driving force $F_{p}$ is always less than the adhesion resistance $F_{adh}$ when its droplets move, i.e., the combined force *F* is less than 0. The direction of the combined force *F* on the droplet is opposite to the direction of its motion. As the surface tension $\gamma$ increases, the droplet receives a larger combined force and the droplet flows more slowly.


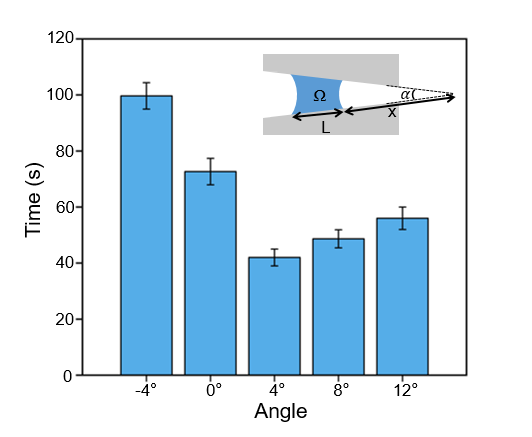


**Figure S18.** Force analysis of the droplet in the suction disc guide channels at an angle greater than 0°.

The difference in curvature between the two ends creates a Laplace pressure difference that moves the droplet towards the apex, where the Laplace differential pressure can be expressed as $P=\gamma/{(\alpha x)}-\gamma/{\alpha(x+L)}$, where $\alpha$ is the angle; *x* is the distance from the droplet to the apex; *L* is the length of the droplet; $\text{γ}$ is the surface tension coefficient of the droplet. When $x\gg L$, the Laplace differential pressure can be expressed as $P={\gamma L}/{\alpha x^{2}}$. So the pressure gradient in the direction of the droplet length is $\text{γ}/{\text{α}\text{x}^{\text{2}}}$. Integrating the pressure gradient over the droplet volume ($\Omega$) gives the Laplace driving force $F_{p}=\int\gamma/{\alpha x^{2}}\cdot\cos\theta d\Omega=\cos\theta\cdot{\pi\gamma\alpha L^{2}}/{2x}={\cos\theta\cdot8\gamma\Omega^{2}}/{\pi\alpha^{3}x^{5}}$.

Supplementary videos

Video S1. Desorption process of the complete device.

Video S2. Desorption process of the no disc channel device.

Video S3. Desorption process of the no UV glue device.

Video S4. Full flow time of UV glue in different suction discs.

Video S5. Fixation process of the bionic fixation device.

Video S6. Carrying an adult weighing 75kg on a rough surface using two devices.
